# The miRNome function transitions from regulating developmental genes to transposable elements during pollen maturation

**DOI:** 10.1093/plcell/koab280

**Published:** 2021-11-10

**Authors:** Cecilia Oliver, Maria Luz Annacondia, Zhenxing Wang, Pauline E Jullien, R Keith Slotkin, Claudia Köhler, German Martinez

**Affiliations:** Department of Plant Biology, Swedish University of Agricultural Sciences and Linnean Center for Plant Biology, Uppsala 75007, Sweden; Department of Plant Biology, Swedish University of Agricultural Sciences and Linnean Center for Plant Biology, Uppsala 75007, Sweden; Department of Plant Biology, Swedish University of Agricultural Sciences and Linnean Center for Plant Biology, Uppsala 75007, Sweden; College of Horticulture and State Key Laboratory of Crop Genetics and Germplasm Enhancement, Nanjing Agricultural University, Nanjing 210095, China; Key Laboratory of Landscaping, Ministry of Agriculture and Rural Affairs and Key Laboratory of Biology of Ornamental Plants in East China, National Forestry and Grassland Administration, Nanjing Agricultural University, Nanjing 210095, China; Institute of Plant Sciences, University of Bern, Bern 3013, Switzerland; Donald Danforth Plant Science Center, St. Louis, Missouri 63132, USA; Division of Biological Sciences, University of Missouri Columbia, Columbia, Missouri 65201, USA; Department of Plant Biology, Swedish University of Agricultural Sciences and Linnean Center for Plant Biology, Uppsala 75007, Sweden; Max Planck Institute of Molecular Plant Physiology, Potsdam-Golm 14476, Germany; Department of Plant Biology, Swedish University of Agricultural Sciences and Linnean Center for Plant Biology, Uppsala 75007, Sweden

## Abstract

Animal and plant microRNAs (miRNAs) are essential for the spatio-temporal regulation of development. Together with this role, plant miRNAs have been proposed to target transposable elements (TEs) and stimulate the production of epigenetically active small interfering RNAs. This activity is evident in the plant male gamete containing structure, the male gametophyte or pollen grain. How the dual role of plant miRNAs, regulating both genes and TEs, is integrated during pollen development and which mRNAs are regulated by miRNAs in this cell type at a genome-wide scale are unknown. Here, we provide a detailed analysis of miRNA dynamics and activity during pollen development in *Arabidopsis thaliana* using small RNA and degradome parallel analysis of RNA end high-throughput sequencing. Furthermore, we uncover miRNAs loaded into the two main active Argonaute (AGO) proteins in the uninuclear and mature pollen grain, AGO1 and AGO5. Our results indicate that the developmental progression from microspore to mature pollen grain is characterized by a transition from miRNAs targeting developmental genes to miRNAs regulating TE activity.

## Teaser

sRNA, PARE, and AGO-IP sequencing uncovered the role of miRNAs during pollen development, showing that miRNAs transition from regulating genes involved in development to transposable elements.

## Introduction

Small noncoding RNAs control essential gene regulatory networks in eukaryotes at the transcriptional, post-transcriptional and translational level. This RNA category includes different classes of small RNAs (sRNAs) that have distinct biogenesis pathways, roles and cellular distribution that (in general) use sequence complementarity to recognize their target RNAs and degrade their transcripts and/or inhibit their translation (Stefani and Slack, 2008). Several technical innovations have enabled researchers to uncover the role of novel classes of sRNAs, their cellular distribution and their roles during different stages of development of an organism or a tissue. According to their origin and biogenesis mechanisms, sRNAs can be classified into microRNAs (miRNAs), small interfering RNAs (siRNAs), piwi-interacting RNAs, or tRNA-derived fragments, among others (for recent reviews on the topic, see, Czech et al. (2018); Martinez (2018); Rosa et al. (2018).

In plants, two classes of sRNAs predominate: siRNAs and miRNAs (Borges and Martienssen, 2015). These two types of sRNAs have different biogenesis pathways and functions. siRNAs are the result of the processing of double-stranded RNAs of diverse origin by Dicer-like proteins (DCL), mainly DCL4, DCL2, and DCL3. This leads to the production of double-stranded sRNAs of 21, 22, and 23/24 nucleotides (nt, produced by DCL4, DCL2, and DCL3, respectively) of which one of the strands will be selectively incorporated into an Argonaute (AGO) protein. Particularly, AGO1 and AGO2 load siRNAs associated with posttranscriptional gene silencing while AGO3, AGO4, AGO6, and AGO9 load siRNAs originated from heterochromatic regions that are associated with RNA-directed DNA methylation (RdDM; Axtell, 2013). On the other hand, miRNAs originate from *MIRNA* genes that produce noncoding transcripts with high self-complementarity that fold into a short hairpin structure that naturally generates a dsRNA without the need of RNA-dependent RNA polymerase (RDR) activity. This hairpin is cleaved by DCL1 into a double-stranded sRNA of 21–22 nts in length. One of these sRNAs will then be selectively loaded into AGO1, AGO7, or AGO10 and form the RNA-induced silencing complex, which uses the sRNA sequence to target mRNAs with perfect or imperfect sequence homology. In plants, this targeting normally leads to the cleavage of the mRNA, but can also induce translational repression or the production of secondary siRNAs (Axtell, 2013; Ma et al., 2013).

Both miRNAs and siRNAs regulate a diversity of processes including development, defense, reproduction, and genome stability. Generally, miRNAs are associated with the regulation of development through the post-transcriptional control of transcription factor mRNAs (Axtell, 2013). Additionally, miRNAs are involved in the generation of secondary siRNAs through the initial targeting of a transcript that stimulates RDR activity leading to the synthesis of a dsRNA that is processed into secondary siRNAs by DCL activity. Secondary siRNAs can target their original transcript or act in trans, targeting other mRNA targets (Axtell, 2013). Secondary siRNAs such as trans-acting siRNAs (tasiRNAs) and phased siRNAs (phasiRNAs) regulate important processes such as organ morphogenesis, developmental timing, and somatic embryogenesis (tasiRNAs; Hunter et al., 2006) or pre-meiotic reproductive development, flowering time, and resistance to biotic and abiotic stresses (phasiRNAs; Liu et al., 2020). Furthermore, miRNAs play a role in genome protection through the initial targeting of transposable elements (TEs) and generation of secondary siRNAs from their transcripts (Creasey et al., 2014a; Surbanovski et al., 2016). Interestingly, one of the miRNAs targeting TEs, miR845, is involved in generating TE-derived siRNAs in the male gametophyte and mediating genome dosage response (Borges et al., 2018). These examples show the complexity of miRNA-mediated processes and highlight their adaptability as regulatory molecules.

The functional versatility of miRNAs is especially important during reproduction, where cells face the duality of carrying out a very specific developmental program while protecting the genome against TE activity. In organisms like zebrafish (*Danio rerio*; Presslauer et al., 2017), mouse (Yuan et al., 2016), *Caenorhabditis elegans* (McJunkin and Ambros, 2014), or *Drosophila melanogaster* (Eun et al., 2013) miRNAs do not only have a differential accumulation pattern in sperm, but play important roles in sperm maturation, fertilization, and post-fertilization events. In *Arabidopsis thaliana*, hypomorphic alleles of *dcl1* and weak alleles of *ago1* have different reproductive abnormalities and reduced seed set (Jacobsen et al., 1999; Kidner and Martienssen, 2005; Nodine and Bartel, 2010), which points to an important role of miRNAs during reproduction in plants. However, little is known about how miRNA activity might shape the transcriptome during pollen development.

Previous reports of Arabidopsis pollen sRNAs have focused on analysis of their accumulation in the mature pollen grain (Grant-Downton et al., 2009; Borges et al., 2011). Here, we analyze in depth the contribution of miRNAs to the sRNA population during the different stages of pollen grain development, their loading into AGO proteins and their target mRNAs. Overall, our work demonstrates that miRNAs involved in epigenetic regulation, like miR845, are enriched and have higher activity at later stages of pollen grain development, and are preferentially loaded in AGO5. In contrast, miRNAs targeting genes involved in development decrease their activity during pollen development. This coincides with increased transcript levels of their target genes at pollen maturity, which are mainly involved in pollen grain germination. In summary, our work demonstrates that miRNAs modulate the transcriptional and epigenetic landscape during pollen grain development and adapt to its transcriptional and epigenetic reprogramming.

## Results

### Dynamic accumulation of miRNAs during pollen development

**Figure 1:**
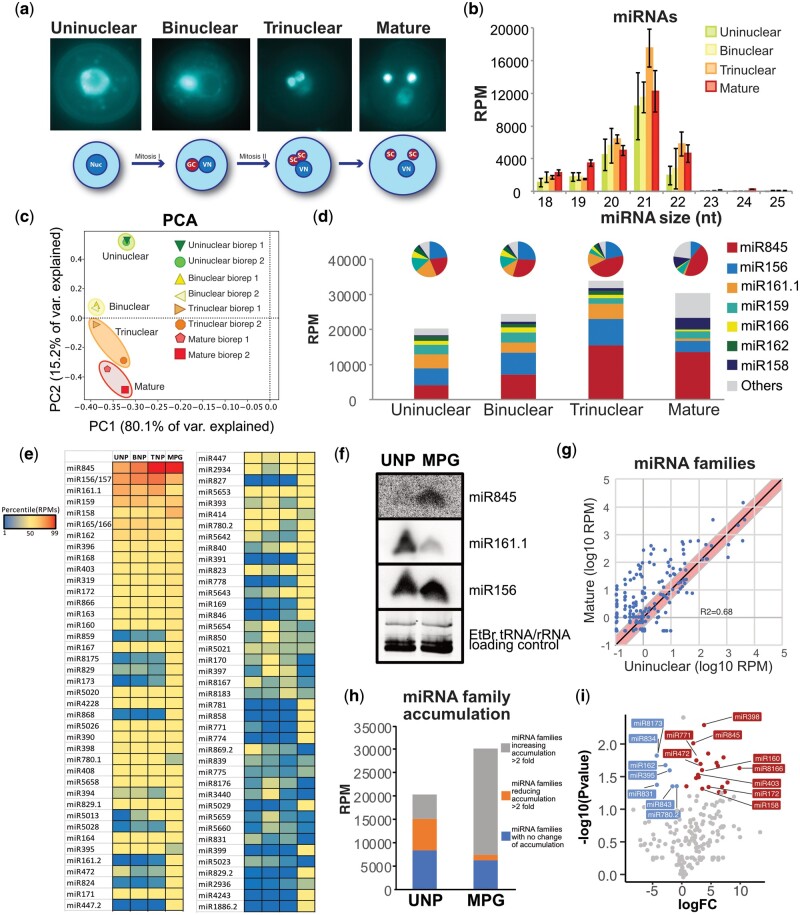
miRNome dynamics during pollen development. A, Representative images of the pollen developmental stages analyzed here. B, microRNA size distribution and accumulation for the developmental stages shown in (A). C, Principal component analysis of the small RNA libraries from different pollen developmental stages based on miRNA levels. D, Bar charts depicting the accumulation of main miRNAs during pollen development for each developmental stage expressed in reads per million. On top of each the same data are shown as a pie chart. E, Heat map of miRNA families accumulation level during pollen development. F, Northern blot showing the increase in accumulation of miR845, the stable accumulation of miR156 and the decrease of miR161.1. The U6 small nuclear RNA was used as a control for RNA loading. 20 μg of total RNA from the uninuclear and mature pollen stages were used to obtain a better resolution of the accumulation dynamic. UNP, uninuclear pollen; MP, mature pollen grain. G, Scatter plot of miRNA family expression values in the uninuclear and mature pollen grain developmental stages. The red line marks a two-fold change in accumulation. H, Categorization of miRNA families according to their increase (more than two-fold, grey), decrease (more than two-fold, orange), or no change (blue) in accumulation in the mature pollen grain compared to the uninuclear pollen. I, Volcano plot of miRNA expression changes between uninuclear and mature pollen grain developmental stages. Significant values (assessed with a paired *t* test, *P* < 0.05) are marked in blue or red for downregulated or overexpressed miRNAs in the mature pollen stage.

To understand the potential sRNA reprogramming during the transition leading to the mature pollen grain, we analyzed sRNA accumulation at four different stages of pollen development (uninuclear, binuclear, trinuclear, and mature pollen grain, representative pictures shown in Figure  1A). Using density centrifugation we isolated four different developmental stages of pollen (Supplemental Figure S1A) as previously described (Dupl'akova et al., 2016). From each different fraction, we isolated total RNA, prepared and sequenced sRNA libraries from two biological replicates for each of the developmental stages (Supplemental Table S1).

We initially focused on analyzing miRNA accumulation due to their important roles both in the regulation of gene and TE transcript abundance, despite accumulating to lower levels during all pollen developmental stages than in other tissues (Supplemental Figure 1B). The total miRNA accumulation profiles between the different developmental stages did not reveal striking differences between the stages (Figure  1B); we only found a slight increase in 21 and 22-nt miRNAs in the trinuclear stage and 22-nt miRNAs in the mature pollen grain in comparison to uni- and binuclear (Figure  1B). Principal component analysis of miRNA family levels in libraries generated from pollen developmental stages demonstrated that biological replicates clustered together (Figure  1C). The analysis of qualitative differences in the miRNA populations between our samples revealed that the majority of miRNA families were present in all our libraries (233 miRNAs); however, we also detected specific miRNAs in each developmental stage, including 23 miRNAs in the mature pollen grain and 13, 7, and 5 in the uni-, bi-, and trinuclear stages, respectively (Supplemental Figure S1C and Supplemental Data Set S1).

We further analyzed the quantitative changes experienced by the most highly accumulating miRNAs (Figure  1, D and E and Supplemental Data Set S2). The comparison between the accumulation level of the top seven miRNA families (representing >90% of all sRNAs in uni-, bi-, and trinuclear pollen and 77% of the mature sRNAs) revealed that there is a striking progressive change in the accumulation of some miRNA families during pollen development. In particular, miR156, miR161.1, miR159, miR166, and miR162, which regulate juvenile-to-adult transitions, defense response and DCL1 (Xie et al., 2003; Zhou et al., 2007; Wu et al., 2009; Guo et al., 2017), maintained or decreased their accumulation at later stages of pollen development (Figure  1, D and E). In comparison, the relative levels of the miRNA families, miR158 and miR845 substantially increased their accumulation (Figure  1, D and E). Specifically, the miR845 family increased until occupying close to 45% of the all sRNAs at pollen maturity (Figure  1D). Although this relative enrichment could be due to a bias in sRNA sequencing (Baldrich et al., 2020), we found a consistent and significant detection of this increase in trinuclear and mature pollen sRNA libraries (Figure  1, D, E, and I). We confirmed this accumulation trend by sRNA gel blot for miR845, miR156, and miR161.1, which increased, maintained, and decreased their accumulation level respectively (Figure  1F;  Supplemental Figure S1, D and E).

Globally, a comparison of the accumulation values between uninuclear and mature pollen indicated that about 34% of miRNA families increased their accumulation, while 66.48% of miRNA families reduced or maintained their accumulation (Figure  1, G and H). However, the contribution of these families to the total miRNome differed, and miRNA families decreasing their accumulation more than two-fold vary during pollen development from a 33% of all miRNA reads in uninuclear pollen to 4% of all reads in mature pollen (Figure  1H). miRNA families with no change of accumulation also slightly decreased during this transition from 42% of all reads in uninuclear pollen to 21% of all reads in mature pollen (Figure  1H). miRNA families with statistically significant increases in accumulation in mature pollen include miR158, miR160, miR172, miR403, and miR845 (Figure  1I). Together, our analysis shows that during pollen development there is a transition of the miRNome from a diverse miRNA pool to a miRNA pool monopolized by miR845.

### AGO1 and AGO5 loading explains miRNA enrichment in the mature pollen grain

**Figure 2:**
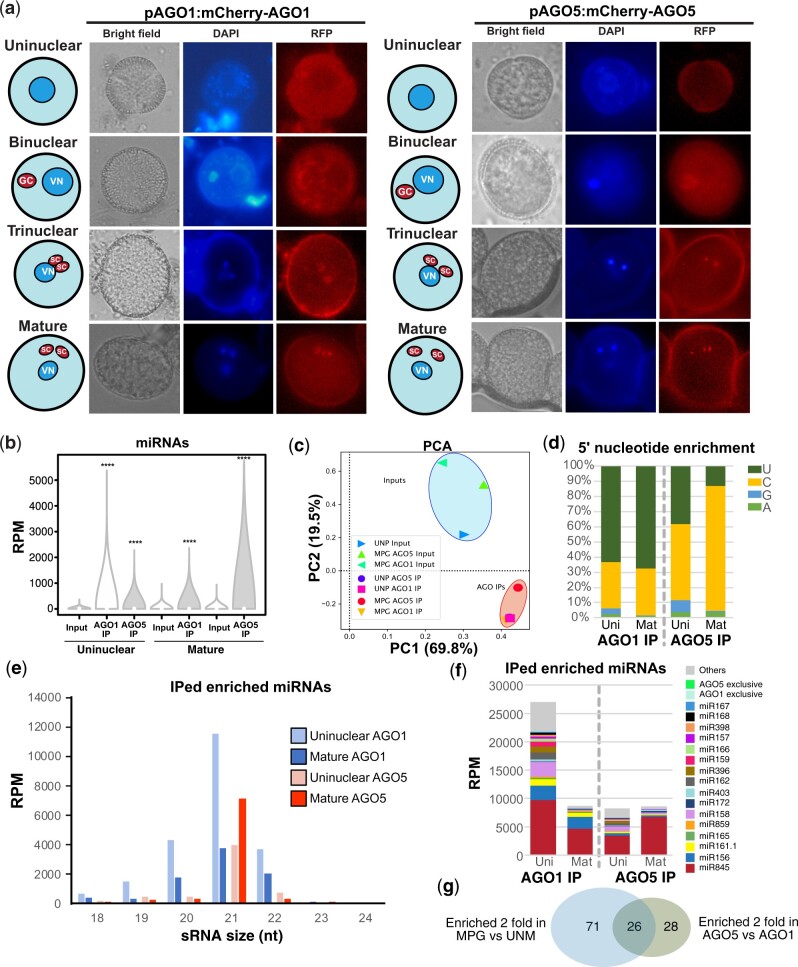
Comparison of AGO1 and AGO5 activity and loaded miRNAs during pollen development. A, Analysis of the cellular localization of AGO1 and AGO5–mCherry–AGO fusion proteins during pollen development. Drawings accompanying the images represent the localization of the different nuclei on each picture. B, Analysis of miRNA enrichment for AGO1 and AGO5 immunoprecipitated sRNAs compared to their respective input controls. Significance was assessed by means of a Mann–Whitney U test (**** denotes a significance of *P* < 0.00001). C, Principal component analysis of the small RNA libraries from different AGO immunoprecipitations and their input controls based on miRNA levels. Venn diagram shows the overlap between the miRNA families loaded in AGO1 and AGO5. D, 5′-nt enrichment for AGO-enriched miRNAs. E, Size distribution of miRNAs enriched in the AGO1 and AGO5 IPs. F, Accumulation of enriched miRNA families in AGO1 and AGO5. In (C)–(F) miRNAs were considered enriched if their accumulation was higher than two-fold their accumulation in the input library or if it was only detected in the AGO immunoprecipitated library. G, Venn diagram showing the overlap of miRNA families enriched more than two fold in the mature pollen grain (blue circle) and miRNA families enriched more than two-fold in AGO5 IP compared to AGO1 IP. Significance (*P* = 0.032807) was calculated using the Chi-square statistic with Yates correction.

miRNA loading into AGO proteins determines their effect at the cellular level (Hutvagner and Simard, 2008). Several AGO proteins have been reported to be active in the Arabidopsis male gametophyte, including AGO1, AGO2, AGO4, AGO5, and AGO9 (Olmedo-Monfil et al., 2010; Borges et al., 2011; Martinez et al., 2016; Oliver et al., 2016), but the sRNA populations loaded into them have not been studied. To shed light into the characteristics of the RISCs in the pollen grain, we analyzed the subcellular localization during pollen development and sRNAs bound to the main miRNA-related AGO proteins expressed in the pollen grain: AGO1 and AGO5 (Supplemental Figure S1, F–H). Previous reports indicated that these two AGOs have different subcellular localization in the mature pollen grain; while AGO1 is located in the vegetative nucleus and in the vegetative cell (VC; McCue et al., 2012), AGO5 accumulates in the sperm cell (SC) cytoplasm (Borges et al., 2011). To understand their role during pollen development we investigated their cellular localization during this process using recently developed reporter lines that functionally complement their respective mutants (Jullien et al., 2020). AGO1 was present already in the cytoplasm of uninuclear pollen and this localization pattern was maintained in the binuclear stage, while it changed at the trinuclear and mature stages, where it is located mainly in the SCs (Figure  2a). On the other hand, AGO5 was only detected in the generative cell (GC) at the binuclear stage and in the SCs at the trinuclear and mature stages (Figure  2A).

Next, to understand their molecular roles in detail, we identified the sRNAs loaded into each AGO in the uninuclear and mature pollen stages by immunoprecipitation of AGO complexes using AGO-specific antibodies followed by sRNA sequencing. In line with their predicted role, we detected enrichment for miRNAs in the immunoprecipitated sRNAs compared to their input controls (Figure  2B;  Supplemental Figure S2, A and B, *P* < 0.00001 tested with a Mann–Whitney *U* test). Principal component analysis of miRNA family levels in libraries generated from AGO immunoprecipitates demonstrated that both AGO-enriched miRNAs clustered together (Figure  2C). Indeed, both AGOs shared a proportion of their respectively enriched miRNAs at the uninuclear and mature stages (59/81% and 73%/85% for AGO1/AGO5 miRNomes at uninuclear and mature stages, respectively, for miRNAs only detected in the immunoprecipitated fraction or enriched more than two-fold respective to the input control, Supplemental Figure S2C). Both AGOs were enriched in miRNAs with different characteristics according to their 5′-nt preference (Figure  2D) and size (Figure  2E). Both at the uninuclear and mature stages AGO5 loads miRNAs of 21 nts in size with a 5′-terminal C, while AGO1 binds miRNAs with a size ranging from 20 to 22 nt with a 5′-terminal U (Figure  2, D and E). This is in line with previous reports of AGO loading preferences in somatic tissues (Mi et al., 2008).

Next, we analyzed the enrichment for miRNA families for each AGO (Figure  2F;  Supplemental Data Set S3). Both AGOs at both pollen developmental stages had a strong preference to load miR845 family members (36/41% and 53/78% of the total miRNome for AGO1/AGO5 at uninuclear and mature stages, respectively). Despite this preferential loading at the uninuclear stage, AGO1 and AGO5 loaded a substantial fraction of miRNAs with well-known roles in the regulation of processes like development, transcription, or RNA silencing. For instance, uninuclear pollen-accumulating AGO1-loaded miRNAs involved in the regulation of different transcriptional and post-transcriptional regulatory factors, including pentatricopeptide (PPR) repeat-containing proteins targeted by miR158 (10%) and miR161.1 (4%), *SQUAMOSA PROMOTER BINDING-LIKE* transcription factors targeted by miR156 (9%), and DCL1 regulated by miR162 (5%; Rhoades et al., 2002; Wu et al., 2009). Mature pollen-accumulating AGO1 increased the accumulation of certain miRNAs like miR156 (25%) and miR161.1 (7%), while reducing the accumulation of the majority of the families (Figure  2F;  Supplemental Data Set S3). On the other hand, uninuclear pollen AGO5 loaded a similar fraction of the miRNAs preferentially loaded by AGO1 at the same developmental stage: miR158 (11%), miR156 (6%), miR162 (5%), and miR161.1 (2%; Figure  2F). At the mature stage, AGO5 experienced a greater decrease in the majority of miRNA families loaded, with only small fractions of miR158 (4%) or miR403 (3%), which regulate, as previously mentioned, PPR proteins and AGO2, respectively (Rhoades et al., 2002; Allen et al., 2005; Figure  2f;  Supplemental Data Set S3). AGO5 is furthermore enriched in miRNAs that are enriched in the mature pollen grain (Figure  2G, *P* = 0.032807) indicating that AGO5-loaded miRNAs might be responsible of the overall miRNome changes observed in the mature pollen grain. The different miRNA-loading pattern might reflect the different roles of both AGOs in relation to their cellular localization, with AGO1 being required to regulate both the developmental program and post-transcriptional activity of TEs in the VC and SCs during the whole extent of pollen development and specially prior to the appearance of the gametic cells (Slotkin et al., 2009; Martinez et al., 2016), while AGO5 might mediate TE control specifically in the GC/SCs (Borges et al., 2018). This also relates to the known role of the VC in controlling the regulation of pollen development, germination and pollen tube growth (Glockle et al., 2018). In summary, AGO1 and AGO5 have different pollen localization patterns and incorporate different populations of miRNAs.

### Inhibition of sRNA activity affects pollen development and germination

To evaluate the level of influence of sRNA activity on pollen grain development and germination, we aimed to inhibit their activity at late stages of pollen development where AGO1 and AGO5 primarily accumulate (Figure  2A). Strong *ago1* and *dcl1* mutants fail to develop gametes (Bohmert et al., 1998; Kidner and Martienssen, 2005; Oliver et al., 2017) making it impossible to analyze their effects during gametophyte maturation. Weak mutants of *ago1* and *dcl1* showed defects in pollen development, with a moderate increase in aborted or shrunken mature pollen grains which was significant for *ago1-27* and *ago5-5* (Supplemental Figure S3A).

**Figure 3:**
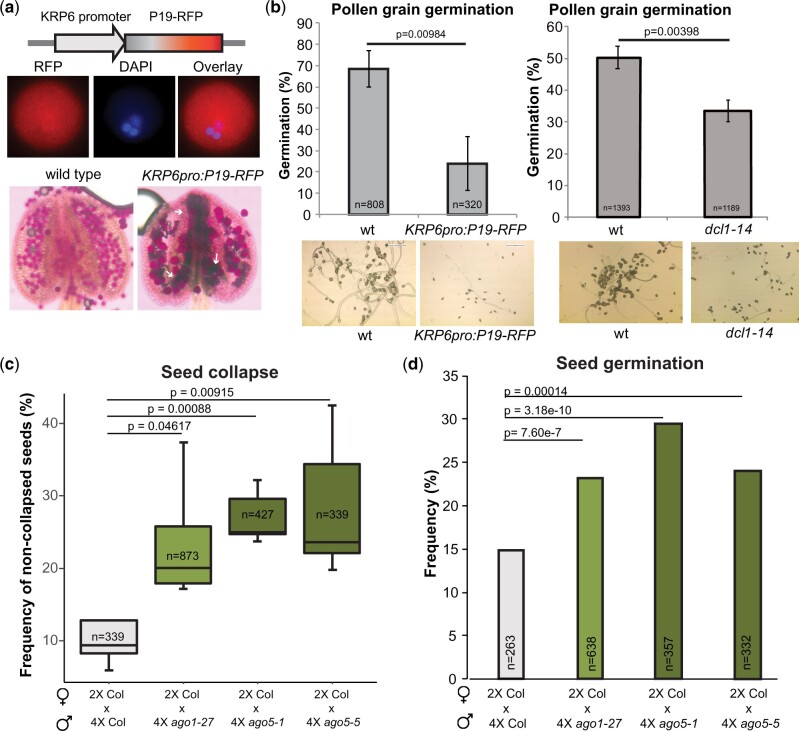
Inhibition of sRNA activity in the pollen grain leads to developmental defects of pollen grain development and inhibition of germination. A, Diagram of the construct used to express the viral silencing suppressor P19 in the mature pollen grain and representative image of its accumulation in mature pollen grains. The lower panel shows representative pictures of the analysis of pollen defects by Alexander staining. Aborted pollen grains are indicated with white arrows. B, Analysis of pollen grain germination in Col-0 wild-type (wt) and *dcl1-14* and wt and KRP6pro:P19-GFP transgenic lines. Representative pictures of each pollen germination experiment are shown. Error bars represent the standard deviation values for the three bioreplicates analyzed. *P*-value is the result of a standard *t* test with two tails and unequal variance. Number of individual pollen grain measurements (*n*) is shown inside of each bar. (C) Frequency of noncollapsed and (D) germinated seeds derived from crosses of wt (2×Col) maternal parents with 4× mutants of indicated genotypes. *t* Test and Chi-squared test were performed in (C) and (D), respectively. Number of individual seed measurements (*n*) is shown inside of each bar. Whiskers in the box plots extend to the maximum and minimum values.

To discern the potential effect of miRNA and/or siRNA activity inhibition without the availability of strong mutant alleles, we hypothesized that the overexpression of a viral silencing suppressor using pollen promoters would drive a cell-specific reduction of AGO1 activity as previously reported (Martinez et al., 2016). The Tombusvirus silencing suppressor P19 is a well-studied protein that inhibits miRNA/miRNA* duplex (Chapman et al., 2004) and siRNA action (Kontra et al., 2016). Accordingly, we expressed P19 (Csorba et al., 2015) fused to red fluorescent protein (RFP) under the control of the *KRP6* promoter to drive the expression at late stages of development of the pollen grain VC (Kim et al., 2008; Martinez et al., 2016; Figure  3A). *KRP6pro::P19-RFP* transgenic lines had defects in pollen development with many of the mature pollen grains aborted at maturity and reduced pollen grain germination (Figure  3, A and B). Furthermore, mutants carrying the weak allele of DCL1, *dcl1-14*, showed a significant decrease in pollen grain germination (Figure  3B). Mutants carrying another weak allele for AGO1, *ago1-52*, displayed abnormal germination phenotypes, with branched pollen tubes (Supplemental Figure S3B), which have not been described in Arabidopsis wild-type pollen although they are common in gymnosperms and certain angiosperm species (Lora et al., 2016). In brief, inhibition of miRNA (and/or siRNA) activity in the male gametophyte VC supports a role of VC sRNAs in the regulation of pollen function.

### AGO1 and AGO5 are required for the triploid block response

Gametic sRNAs establish hybridization barriers in different species (Martienssen, 2010; Senti and Brennecke, 2010). In plants, polyploidization establishes hybridization barriers due to the unbalanced expression of imprinted genes in the endosperm (Kradolfer et al., 2013; Wolff et al., 2015; Huang et al., 2017; Jiang et al., 2017; Wang et al., 2018), in a phenomenon known as the triploid block (Kohler et al., 2010). In Arabidopsis, the triploid block is established upon crosses of a pollen donor forming 2n pollen with a diploid maternal plant. Depletion of paternal sRNAs through mutation of the major Pol IV subunit *NRPD1* or the miRNA gene *MIR845B* suppresses the triploid block response (Erdmann et al., 2017; Borges et al., 2018; Martinez et al., 2018; Satyaki and Gehring, 2019).

To test whether the miRNA populations loaded in AGO1 or AGO5 are responsible for establishing the triploid block, we created tetraploid versions of the AGO1 weak allele *ago1-27* and of the AGO5 alleles *ago5-1* and *ago5-5* and performed crosses with a wild-type diploid mother. The results of these pollinations revealed that paternal tetraploid *ago1-27* weakly, but nevertheless significantly increased triploid seed viability (Figure  3, C and D). Similarly, paternal tetraploid *ago5-1* and *ago5-5* significantly increased triploid seed viability and seed germination (Figure  3, C and D) to a similar level as *ago1*, suggesting that both function in the triploid block response. Thus, paternal AGO1 and AGO5 are part of the triploid block response potentially through their loading of miRNAs.

### miRNA activity against genes is reduced during pollen maturation

To uncover the dynamics of miRNA-regulated transcripts during pollen grain development, we aimed to identify the overall miRNA-targeted mRNAs in the uninuclear, binuclear, and mature pollen grain developmental stages, which represent the most characteristic stages in terms of miRNome diversity and AGO accumulation. To identify miRNA-targets, we made use of the characteristic cleavage activity of plant miRNAs, which leave a fingerprint of their cleavage activity on their target mRNAs (Addo-Quaye et al., 2008; Gregory et al., 2008; German et al., 2009). To that end, we generated and sequenced parallel analysis of RNA ends (PAREs) libraries as previously described (Zhai et al., 2014) from uninuclear, binuclear, and mature pollen grains (Supplemental Table S1). PARE is a technique that enriches AGO protein cleaved mRNAs with a polyA tail but without a 5′-cap for library preparation and sequencing and is conventionally used for miRNA-target identification (Addo-Quaye et al., 2008; Gregory et al., 2008; German et al., 2009). We focus our analysis on 143 high-confidence target sites that were represented in at least two bioreplicates of each of the tissues under study (Supplemental Table S2 and Supplemental Data Set S4).

**Figure 4:**
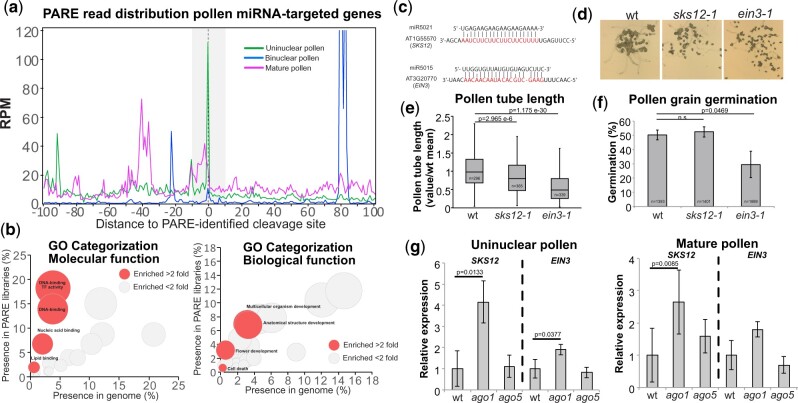
Analysis of miRNA genic targets in the uninuclear, binuclear and mature pollen grain identified by PARE sequencing. A, Distribution of 5′ ends of PARE reads around the predicted cleavage site (located at coordinate 0 in the *X*-axis) in a 100-nt window. Grey zone represents the physical position covered by the bound miRNA. B, Bubble graph depicting the GO term overrepresentation test for molecular (left) and biological (right) function. Bubbles in red indicate GO categories enriched two-fold or more. The diameter of the bubble indicates the number of genes present in that enriched category. C, Examples of two miRNA targets in our PARE analysis: SKS12-miR5021 and EIN3-miR5015. D, Representative pictures of pollen grain germination for wt and the *sks12-1* and *ein3-1* mutants. (E) Length of the pollen tube and (F) percentage of germination for in vitro germinated pollen grains for the genotypes indicated. Number of individual pollen grain measurements (*n*) is shown inside of each bar. G, Analysis of mRNA expression in uninuclear and mature pollen by RT-qPCR for two representative miRNA-targeted genes (*SKS12* and *EIN3*) in wt, *ago1*, and *ago5* mutants. Error bars in (F), (G), and (H) represent the standard deviation values for the three bioreplicates analyzed. *P*-value is the result of a standard *t* test with two tails and unequal variance. Whiskers in the box plots extent to the maximum and minimum values.

miRNAs regulate their target transcripts at the post-transcriptional level by inducing their cleavage and by inhibiting their translation (Yu et al., 2017). In other organisms, mRNAs targeted by miRNAs in the gametes increase their stability (Dallaire et al., 2018). We explored if a similar scenario may apply to Arabidopsis by analyzing the mapping of PARE reads for all miRNA-targeted mRNA reads relative to the miRNA target position. The distribution of 5′-P end reads in a 100-nt window from the identified target sites for all the pollen developmental stages analyzed showed that most of the targeting events resulted in cleavage of the target RNAs (Figure  4A) in opposition to the expected ribosome stalling-characteristic peaks caused by noncleavable miRNA targeting (Hou et al., 2016). Furthermore, miRNA-mediated cleavage was higher in uninuclear pollen compared to binuclear and mature pollen grains (4.39-fold between uninuclear and mature pollen at the predicted miRNA target position, profiles of the bioreplicates shown in Supplemental Figure S4A).

A global categorization of pollen miRNA-regulated transcripts (cleaved by miRNAs in the uninuclear, binuclear, or mature pollen) revealed that the majority are associated with molecular functions related to transcription factor and DNA binding activity and biological functions associated with organ development and growth, reproduction, and genetic/epigenetic regulation (Figure  4B;  Supplemental Table S2). Interestingly, the pollen miRNA-targeted mRNAs included well-studied genes involved both in pollen-associated processes such as *GROWTH-REGULATING FACTOR 1/2/3/8* (*GRF1/2/3/8*), *ARABIDOPSIS TRITHORAX 3* (*ATX3*), *UDP-GALACTOSE TRANSPORTER 1* (*UTR1*), *BASIC LEUCINE_ZIPPER 34* (*AtbZIP34*), and *AUXIN SIGNALING F-BOX 3* (*AFB3*; involved in pollen maturation) or *LOST IN POLLEN TUBE GUIDANCE 1* (*LIP1*) and *MYB101* (involved in pollen tube germination; Cecchetti et al., 2008; Gibalova et al., 2009; Liang et al., 2013; Dong et al., 2015; Chen et al., 2017; Slane et al., 2017; Supplemental Table S2). miRNA-targeted genes increased their expression during pollen development following the opposite trend to miRNA activity (Supplemental Figure S4b). This trend was also the opposite to the majority of transcripts, which decrease their accumulation during pollen maturation (Honys and Twell, 2004). This indicates that there is a distinct and specific miRNA activity during pollen development that regulates genes involved in pollen grain development and germination.

To test the observation that miRNA activity regulates the expression of genes that are important for pollen development, we obtained homozygous mutants for two genes with uncharacterized role in pollen-associated processes that are regulated by miRNAs: *SKU5 SIMILAR 12* (*SKS12*, AT1G55570, mutant termed *sks12-1)* and *ETHYLENE-INSENSITIVE3* (*EIN3*, AT3G20770, *ein3-1*; Kieber et al., 1993; Figure  4C;  Supplemental Figure S4D), which are targeted specifically in pollen by miR5021 and miR5015, respectively (Supplemental Figure S4D and Supplemental Data Sets S4 and S5). We initially evaluated the ability of their mature pollen grains to germinate in vitro (Figure  4D). Measurement of pollen tube length and germination rate after 16 h of incubation indicated that while only *ein3-1* was significantly affected in the rate of pollen germination (Figure  4F), both mutants were significantly impaired in pollen tube growth (Figure  4E), including a second allele of *SKS12* (Supplemental Figure S4C).

To confirm that the activity of these genes is impaired by miRNA activity, we purified uninuclear and mature pollen grains from *ago1-27* and *ago5-1* mutants and analyzed the transcript levels of the target genes *SKS12* and *EIN3* by reverse transcription quantitative polymerase chain reaction (RT-qPCR)*.* The transcript levels of *SKS12* and *EIN3* were significantly increased in *ago1-27* in the uninuclear pollen grain, while only *SKS12* transcripts were significantly increased in the *ago1-27* mature pollen grain (Figure  4G). This result indicates that miRNA activity, potentially modulated by AGO1, regulates transcript levels during pollen development and specially in uninuclear pollen. Thus, we conclude that miRNA cleavage activity (potentially mediated by AGO1) takes place during pollen development and regulates the transcript levels of genes needed for processes that are important for pollen grain development and germination.

### miR845-targeted TEs progressively decrease their level of 24-nt sRNAs during pollen development

**Figure 5:**
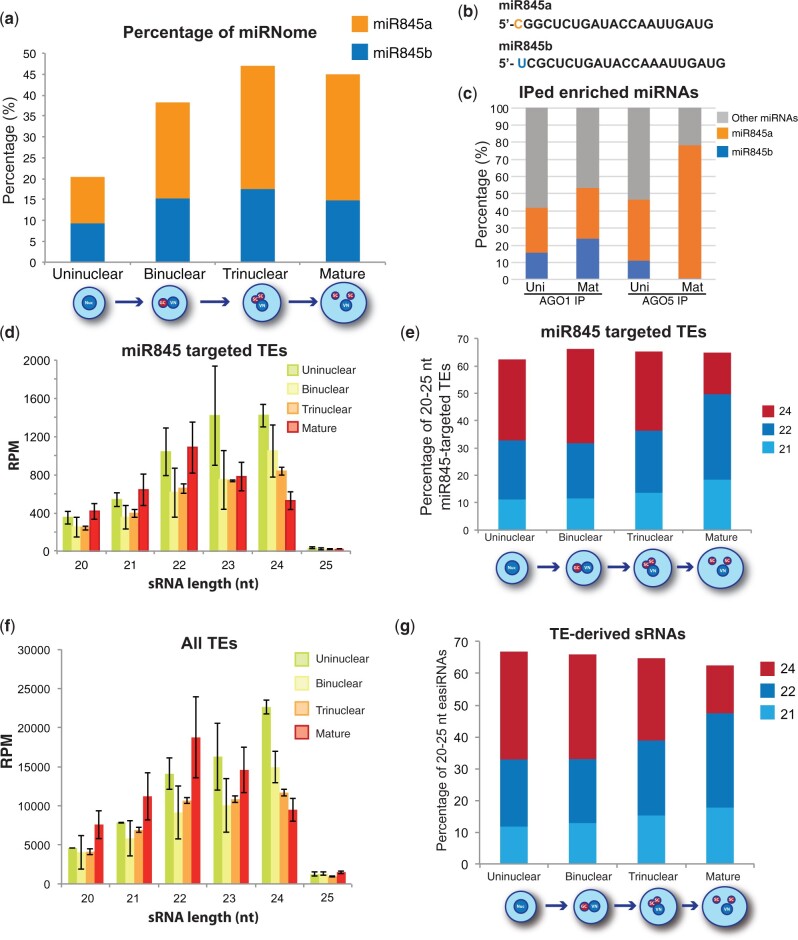
Analysis of miR845 dynamics and its target TEs during pollen development. A, Accumulation percentages for miR845a and b during pollen development. B, Sequence comparison of miR845a and miR845b with the 5′ nucleotide highlighted. C, Percentage of the impact of miR845a and miR845b on total AGO1 and AGO5 immunoprecipitated miRNAs. D, Accumulation size profile of TE-derived siRNAs of predicted miR845-targeted TEs. E, Accumulation of 21-, 22-, and 24-nt sRNAs from miR845-targeted TEs during pollen development shown as percentages of total 20–25-nt sRNAs derived from those TEs. F, Accumulation profile of all TE-derived sRNAs. G, Accumulation of 21-, 22-, and 24-nt sRNAs from TEs during pollen development shown as percentages of total 20–25-nt easiRNAs.

In plants, miRNAs are important post-transcriptional regulators of TEs (Creasey et al., 2014a; Borges et al., 2018). In particular, the miR845 family is involved in the biogenesis of epigenetically active siRNAs (easiRNAs) through the targeting of the primer binding site (PBS) of TEs (Borges et al., 2018). In Arabidopsis the miR845 family is composed by two members, miR845a and miR845b, which are 21 and 22 nt in length, respectively (Borges et al., 2018). Analysis of their presence in our pollen-development sRNA libraries showed that the two members of the miR845 family increased their accumulation, especially miR845a which increased its accumulation by 2.7-fold from uninuclear to mature pollen (Figure  5A). Both miRNAs did not seem to be affected by fluctuations of the processing precision and were of the expected size at all stages of development (Supplemental Figure S5A). Interestingly, the 5′-terminal nucleotide of miR845a and miR845b (C and U respectively, Figure  5B) suggests a preferential loading in AGO5 and AGO1, respectively (Mi et al., 2008). We analyzed if this predicted differential loading was detectable in our AGO1 and AGO5 IP sRNA libraries and, indeed, AGO5 showed a clear preferential loading of miR845a at the mature pollen stage (77% of AGO5-enriched IPed miRNA sequences, Figure  5C).

Members of the miR845 family were proposed to trigger Pol IV-dependent easiRNA biogenesis during meiosis or early gametogenesis (Borges et al., 2018). Analysis of easiRNAs derived from miR845-targeted TEs indicates that easiRNAs accumulated to high levels already at the uninuclear stage (Figure  5D), consistent with recently published work (Wang et al., 2020). As expected from its preferential loading into AGO1, miR845b-targeted TEs produced easiRNAs earlier and to a greater extent than miR845a targets (Supplemental Figure S5B). Interestingly, during pollen grain development, there was a gradual transition from a majority of 24 nt at the uninuclear stage to a majority of 22 nt at pollen maturity (Figure  5, D and E), a feature that was exclusive for TE-derived sRNAs since total sRNAs did not follow this trend (Supplemental Figure S5B).

This tendency of losing 24-nt sRNAs during pollen grain development was common for all TEs and independent of the presence of a miR845 target site (Figure  5, F and G). This might indicate that either all TEs are targeted by a similar mechanism, or that miR845 is able to induce a silencing cascade that extents to the majority of TEs in the pollen grain. To better understand this, we analyzed TE-derived sRNAs in *MIR845B* mutant pollen sRNA libraries (Borges et al., 2018). This analysis showed that TE-derived 21/22-nt sRNAs were reduced when miR845b accumulation was impaired (Supplemental Figure S5C), indicating that miR845b globally affects the generation of 21/22-nt sRNAs from TEs. However, we cannot exclude that a potential degradation of AGO4-24 nt sRNA complexes might be taking place during pollen development biasing the presence of TE-derived 21/22 sRNAs, as recently suggested (Panda et al., 2020). Overall, these results show that TEs tend to lose 24-nt sRNAs while gaining 21/22-nt sRNAs during pollen development in line with an increase of the accumulation of miR845 family members, specially miR845a, which is preferentially loaded into AGO5.

### TE-targeting activity mediated by miRNAs increases during pollen development

**Figure 6:**
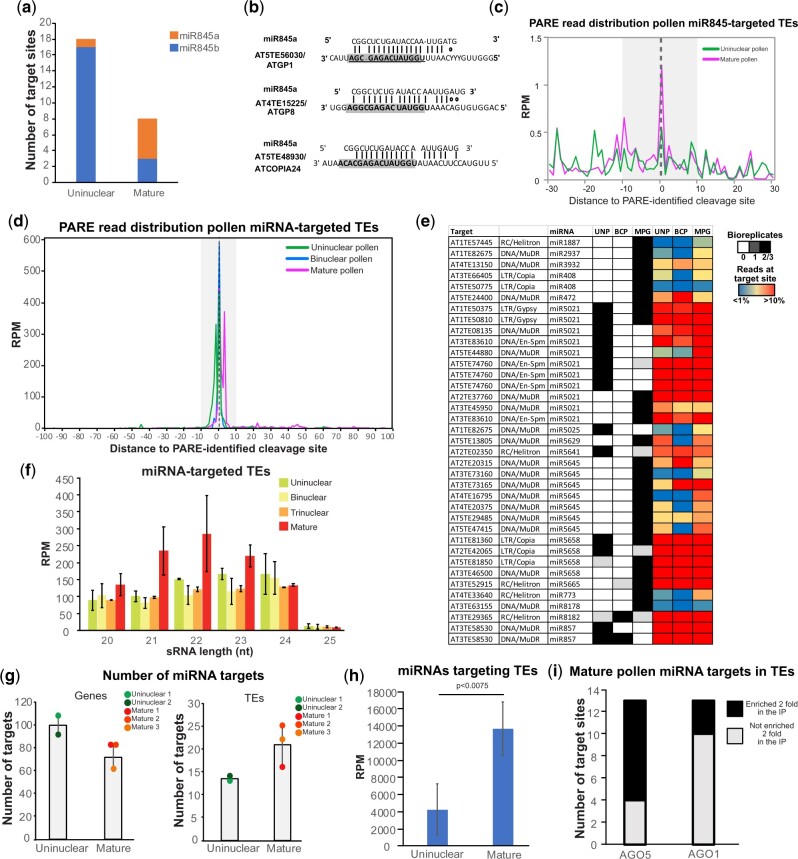
Analysis of miRNA TE targets in the uninuclear, binuclear, and mature pollen grain identified by PARE sequencing. A, Number of miR845a- and miR845b-targeted TEs identified in uninuclear and mature pollen grain PARE libraries using high-confidence parameters. B, Representative examples of miR845a targeting positions in TEs. The location of the two different PBSs is highlighted in gray. C, Distribution of 5′-ends of PARE reads around the confirmed miR845-targeted TEs cleavage site (located at coordinate 0 in the *X*-axis) in a 30-nt window. Gray zone represents the physical position covered by the bound miRNA. D, Distribution of 5′-ends of PARE reads around the confirmed miRNA-targeted TEs cleavage site (located at coordinate 0 in the *X*-axis) in a 100-nt window. Gray zone represents the physical position covered by the bound miRNA. E, High-confidence identified miRNA-targeted TEs in PARE libraries. Target TE and its associated miRNA are indicated together with a heatmap of the presence of the target site in the different number of unicellular or mature PARE libraries in different shades of gray, and the level of accumulation of PARE reads in a 20-nt window surrounding the miRNA target site compared to the total accumulation of PARE reads for that gene for each individual bioreplicate studied with a blue-red gradient. F, Accumulation size profile of TE-derived siRNAs of PARE identified miRNA-targeted TEs. G, Number of targeted genes (left) and TEs (right) in uninuclear and mature pollen PARE libraries. Values for each bioreplicate are shown. H, Total accumulation values (in reads per million) of TE-targeting miRNA families (shown in (B) and including miR845) in uninuclear and mature pollen sRNA libraries. *P*-value was obtained using a paired *t* test. I, Number of identified TE-miRNA target sites in the mature pollen grain enriched more than two-fold (black) or not enriched (white) in AGO5 and AGO1.

Next, we analyzed miRNA activity against TEs during pollen development. Using the same stringent parameters used for genic miRNA targets, miR845 activity was identified in individual bioreplicates. As expected from the higher accumulation of miR845a in the mature pollen grain, we identified a higher number of targeting events in the mature pollen compared to uninuclear pollen (Figure  6A). These targeting events include the nonstandard target position of miR845 at the tRNA-Met PBS of several ATGP and ATCOPIA elements as previously predicted (Borges et al., 2018; Figure  6B;  Supplemental Data Sets S6 and S7 and Supplemental Figure S6, A–C). Using similar target prediction parameters as Creasey et al. (2014b) allowed us to identify a higher number of TEs targeted by the miR845 family in our PARE libraries compared to more stringent parameters (Supplemental Data Set S8 including several TEs previously predicted by Borges et al. (2018)). Analysis of the distribution of PARE reads for these miR845-targeted TEs indicated that miR845 cleavage activity was slightly higher in the mature pollen compared to the uninuclear pollen (2.3-fold higher on average at the predicted target site, Figure  6C replicates shown in Supplemental Figure S6A).

Following the same stringent detection parameters used for the detection of genic miRNA targets (target sites that were represented in at least two bioreplicates of each of the tissues under study), we identified 31 miRNA-targeted TEs (37 miRNA-targeting events, Figure  6, D and E), strongly suggesting that Pol II transcribes these TEs (since they are detected by PARE sequencing). Interestingly, these TEs were targeted mostly by pollen-specific miRNAs like miR5021, miR5658, and miR5645 (Figure  6E), which were enriched or only detected in AGO1 and AGO5-IPed sRNA libraries from uninuclear pollen grains (miR5021, miR5645, and miR5658) and/or identified also in AGO5-IPed sRNAs from mature pollen grains (miR5021 and miR5658; Supplemental Data Set S3).

To confirm that the activity of these TEs is impaired by miRNA activity, we purified uninuclear and mature pollen grains from *ago1-27* and *ago5-1* mutants and analyzed by RT-qPCR the transcript levels of the TEs *AT5TE48930* (targeted by miR845) and *AT5TE81850* (targeted by miR5658)*.* The transcript levels of the two miRNA-targeted TEs were significantly increased in *ago5-1* in the uninuclear pollen grain, and additionally *AT5TE48930* was significantly increased in *ago5* mature pollen grain (Supplemental Figure S6B). This indicates that miRNA activity, potentially mediated by AGO5, regulates TE transcript levels.

miRNA-targeted TEs produced high levels of 21/22-nt sRNAs even at the uninuclear stage, but their maximum production took place at the mature stage (Figure  6F), revealing a potential increased activity of miRNAs against this group of TEs at pollen maturity. Supporting the notion that this group of TEs is bonafide miRNA targets, their sRNA production decreased in *dcl1*, *dcl2/4*, and *dcl1/2/4* (Supplemental Figure S6D). This group of TEs did not show differences in their DNA methylation levels compared to the rest of TEs (Supplemental Figure S6E), suggesting that miRNA targeting does not seem to have consequences for their DNA methylation levels. Altogether, our data point to the existence of Pol II-transcribed TEs during pollen epigenetic reprogramming that are regulated by miRNAs more actively in the mature pollen grain.

Finally, to understand the dynamics of miRNA targeting of TEs during pollen development, we studied in detail their accumulation patterns in our sRNA libraries from AGO immunoprecipitates at different stages of pollen development. High-confidence TE target sites increase their presence in mature pollen compared to uninuclear pollen, while the opposite happens for genic target sites (Figure  6G). This indicates that the activity of miRNAs against TEs is higher in the mature pollen grain. In parallel with the increase of targeting events in TEs, miRNAs targeting TEs increased their accumulation during pollen development (Figure  6H, *P* < 0.0075). Furthermore, miRNA target sites present in TEs in mature pollen belonged mainly to miRNAs that were enriched at least two-fold in mature pollen grain-accumulating AGO5 (69.2% of all target sites, Figure  6I). All these lines of evidence indicate that AGO5 accumulation in the mature pollen SCs might influence the global population of miRNAs and lead their activity against TEs in the gamete cells. In summary, our data show that during pollen development there is a switch of miRNA activity towards increased TE-targeting in the mature pollen grain that is mainly mediated by miRNAs loaded into AGO5.

## Discussion

**Figure 7:**
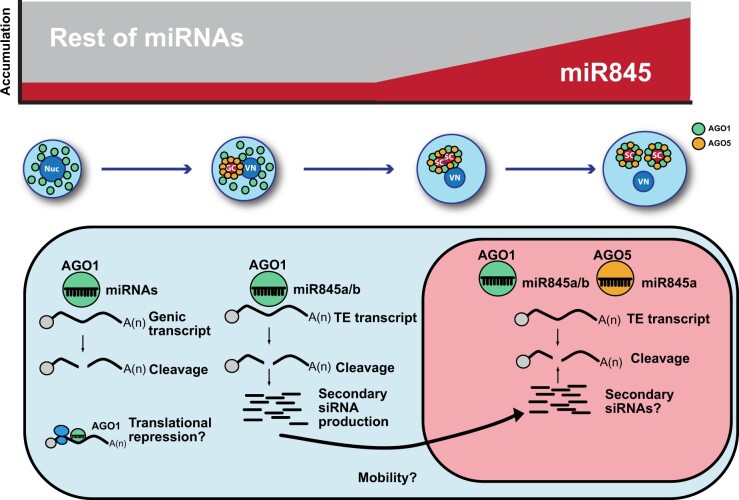
Model for miRNome changes during pollen development. miRNA populations change during pollen development and are influenced at maturity by the increased presence of miR845, in line with the increased accumulation of AGO5. The lower part shows the distinct functionality of the two main miRNA effectors, AGO1 and AGO5. While AGO1 is able to target both genes and TEs, AGO5 mainly targets TEs due to its preferential loading of miR845a. Furthermore, AGO1 loading of miR845b (which is 22 nts in length) might induce the production of mobile TE-derived siRNAs. These secondary siRNAs might help to target other TEs in the SCs or might inhibit the establishment of DNA methylation via targeting of Pol IV transcripts.

The pollen grain undergoes transcriptional and epigenetic reprogramming during its transition to maturity, but whether the first is a consequence of the latter is unknown (Honys and Twell, 2004; Slotkin et al., 2009; Calarco et al., 2012; Feng et al., 2013). Through the use of a technically challenging protocol for pollen developmental stage separation combined with high-throughput sRNA sequencing, PARE sequencing from uninuclear, binuclear and mature pollen grain pollen developmental stages and AGO immunoprecipitation followed by sRNA sequencing from uninuclear and mature pollen grains we have: (1) identified the characteristics of the miRNome during pollen grain development; (2) determined the miRNA populations loaded into the main AGO proteins in the pollen grain, AGO1 and AGO5; (3) identified miRNA targets (both TEs and genes); and (4) identified the involvement of both AGO1 and AGO5 in the triploid block. Our data reveal that the miRNome experiences a transition during pollen development from a miRNome involved in the control of transcripts associated with pollen development and germination to a miRNA population focused on the post-transcriptional control of TEs (Figure  7).

This duality of miRNA activity is highlighted in our work through sRNA sequencing and identification of miRNA-targeted mRNAs through PARE sequencing, which show their importance in the regulation of both genes and TEs (Figures  4 and 6). Interestingly, our PARE and sRNA data indicate that these two activities are partially restricted during pollen development to the uninuclear pollen (gene regulation) or mature pollen (TE regulation).

During pollen maturation, genes targeted by miRNAs (identified by PARE sequencing) were functionally associated with processes related with pollen development and germination and increased their expression in the mature pollen stage (Figure  4B;  Supplemental Figure S4B). This could be explained by two different mechanisms: (1) miRNAs increase the stability of their target transcripts, as previously observed in *C. elegans* (Dallaire et al., 2018) or (2) miRNA activity against genes is higher at the uninuclear stage and is reduced in the mature pollen grain. According to our data we favor the second mechanism since several pieces of evidence indicate that miRNA activity is higher in the uninuclear pollen: (1) the miRNome is more diverse in the uninuclear pollen (Figure  1D); (2) reduction of miRNA activity using a weak *dcl1* allele or via the expression of the P19 viral silencing suppressor in the VC reduced pollen grain viability and germination (Figure  3, A and B); (3) PARE data indicate that miRNA targeting events induced the cleavage of their target mRNAs mainly in the uninuclear pollen (Figure  4A); and (4) transcript levels of selected miRNA targets affecting pollen development (*SKS12* and *EIN3*) were upregulated in an *ago1* mutant mainly in the uninuclear pollen (Figure  4G). Furthermore, AGO1 localization and miRNA activity in the VC at the uninuclear and binuclear stages are in line with the known role of the VC in the regulation of pollen development and germination, which is independent of the presence of SCs (Glockle et al., 2018).

In summary, our data show that during pollen development miRNA activity against genes is more active in the uninuclear pollen, affects genes important for pollen development and germination and is probably regulated by AGO1 in the VC. Furthermore, our PARE sequencing analysis indicates that miRNA gene-targeting activity decreases in mature pollen (Figure  4A;  Supplemental Table S2). This decrease of activity might be linked to the accumulation of AGO1 in the SCs at later stages of pollen development. Additionally, our PARE analysis identified several components of the miRNA pathway that are regulated by miRNAs and have activity in the mature pollen grain including AGO1 (miR168), AGO2 (miR403), and RS41 (miR447). We hypothesize that miRNA gene-targeting role could also be self-regulated by the miRNA pathway itself. However, we cannot exclude that, by an unknown mechanism, miRNA activity mediated by AGO1 switched from inducing cleavage of their target mRNAs in uninuclear pollen to induce their translational repression in the mature pollen grain as previously demonstrated (Grant-Downton et al., 2013) and/or that RNA degradation is very active in the mature pollen grain, which would hide the presence of miRNA-mediated cleavage events in PARE libraries. Nevertheless, our data might indicate that higher miRNA activity in uninuclear pollen could serve to avoid premature expression of important pollen germination or maturation genes in the unicellular stage, in a similar way to the role of miRNAs in embryo development (Nodine and Bartel, 2010). Our work has also identified a number of biological processes and candidate genes associated with pollen development that provide a platform for future reverse genetic studies.

Additionally, we have explored the influence of miRNAs on the regulation of TEs in the pollen grain. During pollen grain development the miR845 family members (miR845a and miR845b) increase in abundance (Figure  1D). This increase likely results in the preferential loading of miR845a into AGO5, a highly abundant AGO protein in the SCs (Figures  2, A and 5, C). miR845 members have been proposed to target Pol IV transcripts of several retrotransposons and induce the production of 21/22-nt easiRNAs from those transcripts (Borges et al., 2018; Martinez et al., 2018). Our analysis demonstrates that indeed simultaneous to the increase in the accumulation of miR845 members during pollen development, there is a parallel decrease of 24-nt sRNAs from their targets and a progressive increase of 21/22-nt sRNAs (Figure  5, D and E), potentially a consequence of their targeting. Indeed, our PARE analysis identified several TEs targeted by miR845a and miR845b, which have higher activity in mature pollen (Supplemental Data Set S6; Figure  6, D and F). Furthermore, PARE-identified TEs that are miRNA targets have high levels of accumulation of 21/22-nt sRNAs, which are at a maximum level in the mature pollen grain (Figure  6C). Interestingly, the preferential loading of miR845 by AGO5 correlates with general low levels of CHH methylation of TEs in the SCs (Calarco et al., 2012). Due to the detection of miR845 activity via PARE sequencing, we speculate that miR845 increased presence in the SCs could be a way to regulate transcriptional reactivation of TEs in the gametic cells. Alternatively, due to the proposed role of miR845 in targeting Pol IV transcripts (Borges et al., 2018), it is also plausible that the increased presence and activity of this miRNA in the SCs upon AGO5 loading might also impair CHH methylation establishment. Interestingly, both AGO1 and AGO5 are required for establishing the triploid block-induced seed collapse (Figure  3, C and D), which might be the consequence of their redundant ability to load miR845 family members.

Together with this, our PARE sequencing and analysis has identified a series of TEs transcribed by Pol II and regulated by miRNAs, which induce the production of 21/22-nt sRNAs from their transcripts (Figure  6, D–F). We speculate that miRNA-targeting of these TEs might be a safeguard mechanism counteracting spurious TE expression (Supplemental Figure S6B). Alternatively, or in parallel, miRNA targeting might produce TE-derived secondary sRNAs, which could be transmitted from the VC to the SCs (Martinez et al., 2016), to protect the SCs against spurious expression of TEs or to target Pol IV transcripts and maintain low levels of CHH methylation in the SCs. Our analysis also highlights an interesting fact about miRNA activity in the pollen grain: while AGO5 loads preferentially 21-nt miRNAs, AGO1 can load miRNAs ranging from 20 to 22 nt in length (Figure  2E). miRNAs of 22 nt are known to induce the production of secondary sRNAs (Chen et al., 2010; Cuperus et al., 2010), which are the class of sRNAs identified as mobile between the VC and the SCs (Martinez et al., 2016). It is plausible to speculate that this relaxation on the size selection of miRNAs by AGO1 leads to a production of mobile secondary sRNAs.

In summary, our work highlights the relevance of miRNAs for the developmental and epigenetic events that occur during pollen grain development in Arabidopsis. The pollen grain needs to face the duality of organizing the transcriptome and epigenome of the newly established gametes in the SCs, while accomplishing a complex developmental program that culminates in the germination of the pollen tube and the successful transfer of the male gametes to the female gametophyte. In mouse and human cell lines, changes in DNA methylation and miRNAs are an important part of the reprogramming of cells to pluripotency (Anokye-Danso et al., 2011; Lin et al., 2011; Miyoshi et al., 2011; von Meyenn et al., 2016), which might be linked to a potential miRNA control of DNA methylation, cell cycle transitions, and regulation of apoptosis (Fabbri et al., 2007; Leonardo et al., 2012). Our data show that during the maturation of the Arabidopsis male gametophyte the main AGO proteins AGO1 and AGO5 are responsible for the regulation of gene (AGO1) and TE (AGO1 and AGO5) transcript levels (Figure  7). The lack of activity of miRNAs against gene transcripts and the appearance of AGO5 at later stages of pollen development (with a preferential loading of miR845a and other miRNAs that shape the mature pollen grain miRNA profile, Figure  2G) explain the switch of the miRNome toward TE control (Figures  2, G and 6, D, H, I). Hence, our work provides insights that the complexity of the orchestration of the miRNome is not exclusive of mammalian reprograming for pluripotency, but also takes place during reproductive development in plants.

## Materials and methods

### Plant material

*Arabidopis thaliana* plants were grown under standard long-day conditions (16-h light and 8-h dark) in a growth chamber illuminated with fluorescent tubes (Philips F25T8/TL841 25 watt tubes with a light intensity of 130 μmol m^−2^ s^−1^) at 22 °C. Plants were sown into potting soil (S-Jord, Hasselfors Garden). The mutant alleles used in this study were *ein3-1* (NASC accession number: N8052), *sks12-1* (SALK_061973), *sks12-2* (SALK_009750), *ago5-1*, *ago5-5*, *ago1-52* (Ler background), and *ago1-27.* The *KRP6pro:P19-RFP* transgene construction, plant transformation and selection were performed as described in Martinez et al. (2016). Primers used for cloning are shown in Supplemental Table S3.

### Total RNA, sRNA RNA gel blot, AGO immunoprecipitation, and sRNA/PARE library construction

Total RNA was isolated using TRIzol reagent (Life Technologies). For miRNA RNA gel blot detection 20 μg of total RNA were loaded in each lane for pollen developmental stage RNA gel blots. sRNA gel electrophoresis, blotting, and cross-linking were performed as described in Pall and Hamilton (2008). The AGO1 and AGO5 proteins were immunoprecipitated using commercially available polyclonal AGO1 (AS09 527) and AGO5 (AS10 671) antibodies (Agrisera AB). AGO immunoprecipitated sRNA libraries were constructed as indicated in McCue et al. (2012) adapted to pollen tissue. PARE libraries were constructed following the protocol described in Zhai et al. (2014) adapted to pollen tissue and using mRNA enriched fractions obtained with the NEB Magnetic mRNA Isolation Kit. All sRNA libraries were made using the NEBNext Small RNA Library Prep Set for Illumina (New England Biolabs) following the manufacturer’s instructions and using gel-enriched sRNAs as described in Martinez et al. (2016).

### Pollen grain separation, germination, viability test, and microscopy

Pollen grain separation was performed as described in Dupl'akova et al. (2016). The pollen developmental stages used for sRNA sequencing correspond to the fractions termed B1 (Uninuclear), B3 (Binuclear), and A3 (Trinuclear). Pollen germination was determined using the media recipe from Rodriguez-Enriquez et al. (2013). Each germination assay was performed in triplicates. Standard Alexander staining method was used to visualize pollen grain abortion as described in Alexander (1969). Visualization of pollen grain germination and Alexander stained pollen grains were performed in a Leica DM RX microscope. For pollen grain fluorescence, pollen grains of T3 plants were mounted on slides containing 50% glycerol and analyzed under a Zeiss Axioplan or a Leica DMI 4000 microscope fluorescence microscopes.

### Bioinformatic analysis

sRNA libraries were trimmed using Trim Galore. Reads were aligned using bowtie (Langmead et al., 2009) with the command “bowtie –q –t –v2” that allows two mismatches. The TAIR10 version of the Arabidopsis genome and the miRbase version 21 were used in this analysis. Reads were normalized to reads per million to the total reads mapped to the Arabidopsis chromosomes. Principal component analysis was performed using the plotPCA tool from deepTools (Ramirez et al., 2016) through the Galaxy platform (Afgan et al., 2018). For PARE library analysis, miRNA cleavage events were identified using PARESnip (Stocks et al., 2018). Identification of high-confidence target sites was performed using astringent PARESnip criteria and considering the presence of such target site in at least two bioreplicates in either uninuclear, binuclear, or mature PARE libraries. For genome-wide plots of PARE reads, PARE libraries were aligned using bowtie and allowing two mismatches in the alignment (-v2). Only confirmed targets on individual bioreplicates were used for the generation of the plots, which represent the average values (in reads per million) from the different bioreplicates.

### Accession numbers

Accession numbers of discussed miRNA-targeted genes are provided in Supplemental Data Set S4. Accession numbers for miRNA-targeted TEs identified in this work are provided in Supplemental Data Sets S6 and S8. Accession numbers of public datasets analyzed in this work are provided in Supplemental Table S4.

## Supplemental data

The following materials are available in the online version of this article.

**Supplemental Figure S1.** Overview of miRNA activity during pollen maturation.

**Supplemental Figure S2.** Analysis of the sRNA populations loaded into AGO and AGO5 at uninuclear and mature stages.

**Supplemental Figure S3.** Characterization of pollen phenotype in mutants for different components of miRNA pathway.

**Supplemental Figure S4.** Overview of miRNA gene-cleavage activity during pollen maturation.

**Supplemental Figure S5.** Analysis of miR845 family members and target TEs during pollen development.

**Supplemental Figure S6.** Overview of miRNA TE-cleavage activity during pollen maturation.

**Supplemental Table S1.** Libraries produced in this study.

**Supplemental Table S2.** High-confidence identified miRNA-targeted genes in PARE libraries.

**Supplemental Table S3.** Primers used in this study.

**Supplemental Table S4.** Publicly available data analyzed in this study.

**Supplemental Data Set S1.** Common and tissue specific miRNAs identified in this study.

**Supplemental Data Set S2.** Accumulation value of miRNA families during pollen development (values expressed in reads per million).

**Supplemental Data Set S3.** miRNA families identified and enriched in AGO1 or AGO5 immunoprecipitates in uninuclear and mature pollen grains.

**Supplemental Data Set S4.** Pollen miRNA-targeted genes identified by PARE sequencing.

**Supplemental Data Set S5.** Accumulation of PARE reads at miRNA target sites.

**Supplemental Data Set S6.** Pollen miRNA-targeted TEs identified by PARE sequencing.

**Supplemental Data Set S7.** Accumulation of PARE reads at miRNA target sites for TEs.

**Supplemental Data Set S8.** Pollen miR845 family-targeted TEs identified by PARE sequencing using parameters similar to Creasey et al. (2014b).

## Supplementary Material

koab280_Supplementary_Data

## Notes

G.M. performed the experimental design. C.O., M.L.A., Z.W., and G.M. performed experiments. P.E.J. and R.K.S. contributed resources. C.K. contributed experimental data. G.M. wrote the manuscript. All authors read and commented on the manuscript.

The author responsible for distribution of materials integral to the findings presented in this article in accordance with the policy described in the Instructions for Authors (https://academic.oup.com/plcell) is: German Martinez (german.martinez.arias@slu.se).
