# Supplementary material for: The miRNome function transitions from regulating developmental genes to transposable elements during pollen maturation
Source: Plant Cell. 2021 Nov 10;34(2):784–801. doi: 10.1093/plcell/koab280 (PMC8824631; doi:10.1093/plcell/koab280)
Supplement: koab280_Supplementary_Data [file koab280_supplementary_data.zip › tpc.21.00529_Supplemental_Figures_and_Tables.pdf]

Supplemental Figure S1

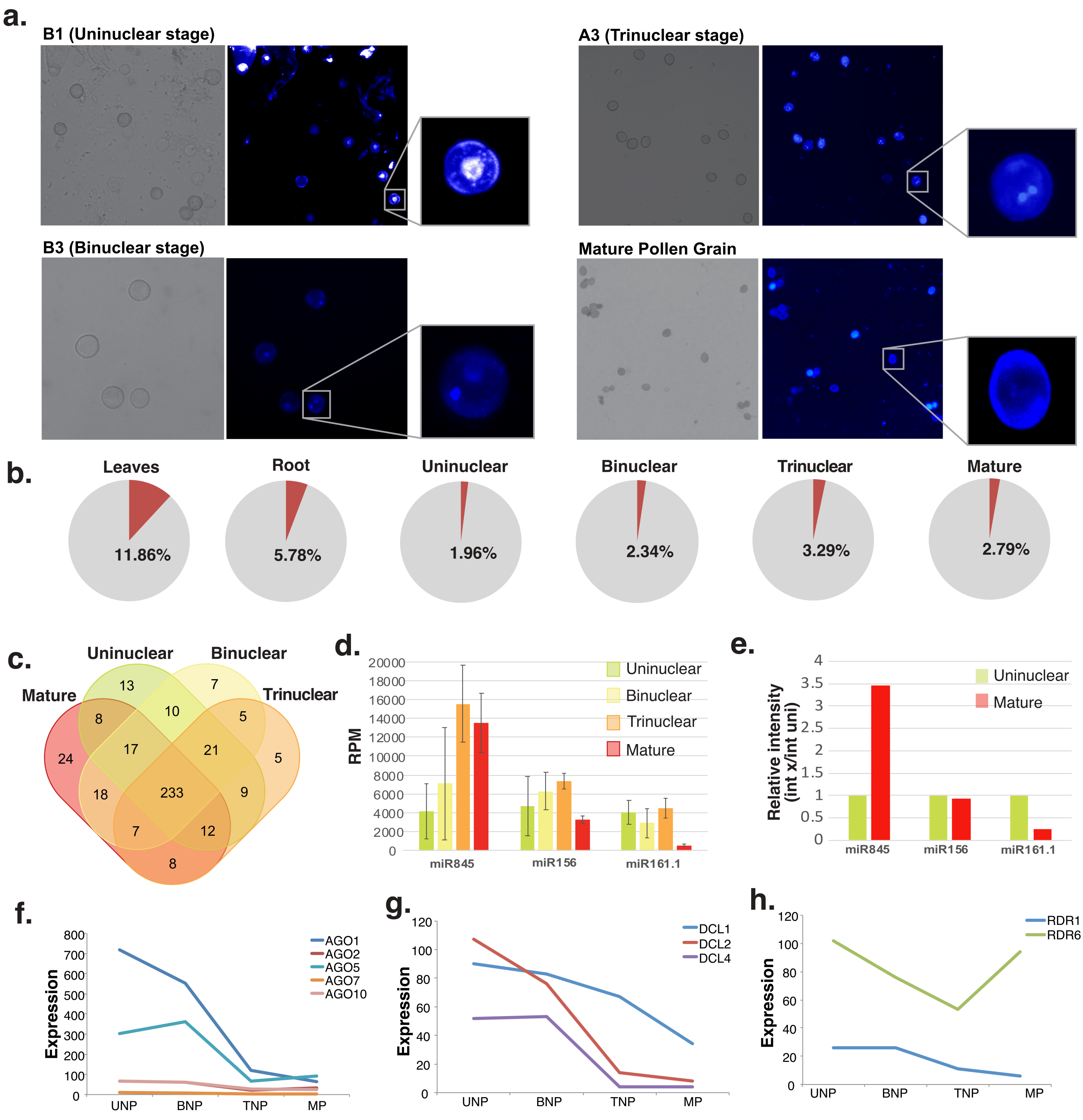

Supplemental Figure S1. Overview of microRNA activity during pollen maturation. Supports Figure 1. a) Representative pictures for each of the fractions of pollen developmental stages analyzed by sRNA high-throughput sequencing: B1 (Uninuclear), B3 (Binuclear), A3 (Trinuclear) and mature pollen grains. b) Percentage of miRNA presence relative to all sRNAs mapped in different Arabidopsis tissues. c) Venn diagram showing the common and developmental stage-specific miRNAs for the stages indicated. d) Accumulation values in the sRNA sequencing libraries for the miRNAs analyzed by RNA gel blot in Figure 1e. e) Quantification of band intensity for the RNA gel blot shown in Figure 1f. f-h) Expression pattern of different RNA silencing components involved in miRNA-related pathways during pollen development: f) AGO, g) DCL and h) RDR genes. Data extracted from ATH1 microarray. UNP= uninuclear pollen, BNP=binuclear pollen, TNP= trinuclear pollen and MP= mature pollen grain

Supplemental Figure S2

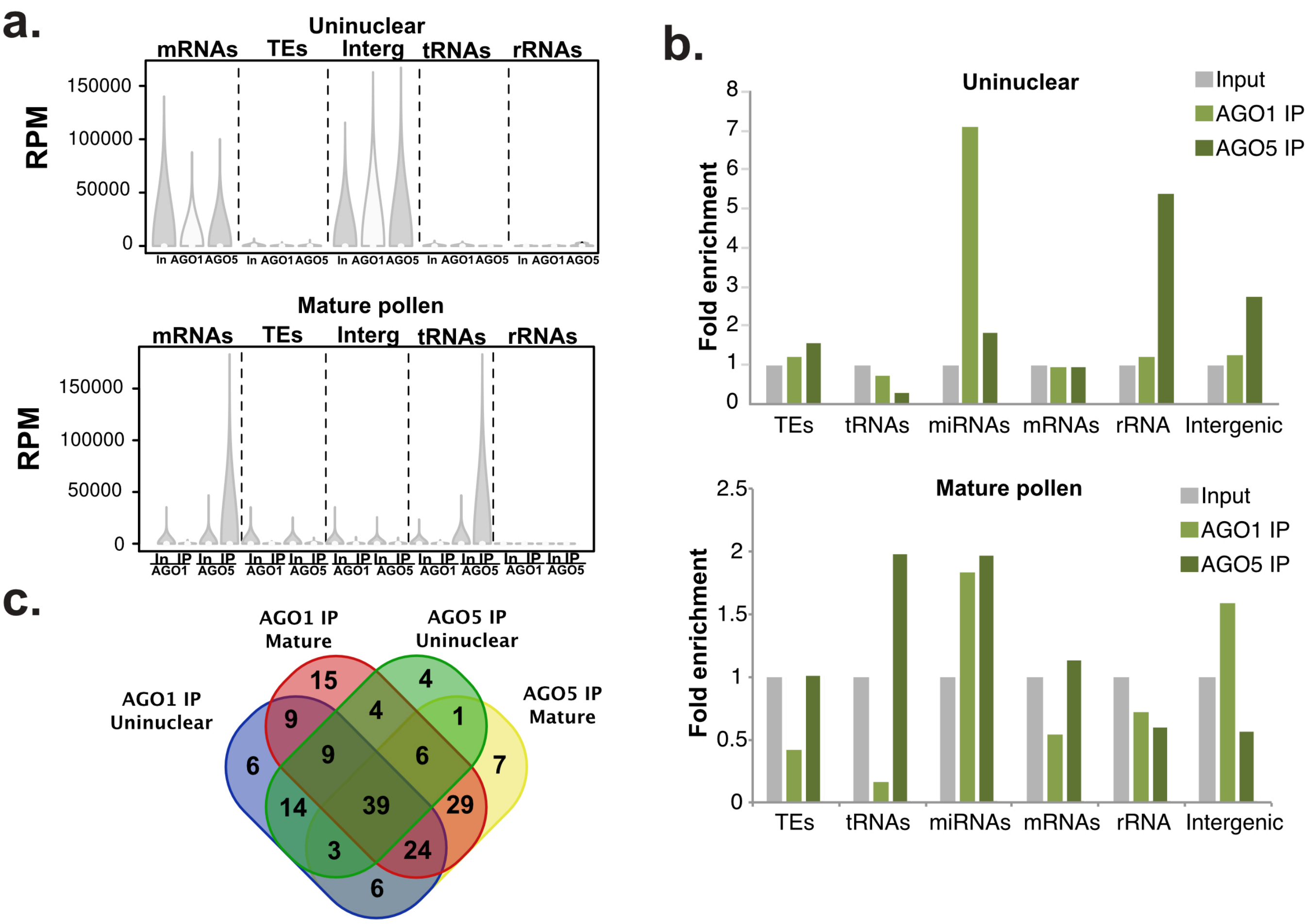

Supplemental Figure S2. Analysis of the sRNA populations loaded into AGO and AGO5 at uninuclear and mature stages. Supports Figure 2. a-b) Analysis of the enriched categories for sRNAs between 18 and 28 nts for AGO1 and AGO5 immunoprecipitated sRNAs compared to their respective input control in a violin plot format (a) or bar chart (b). c) Venn diagram showing the overlap of miRNA families enriched more than two fold in the AGO1 and AGO5 IPs from uninuclear and mature pollen grains.

Supplemental Figure S3

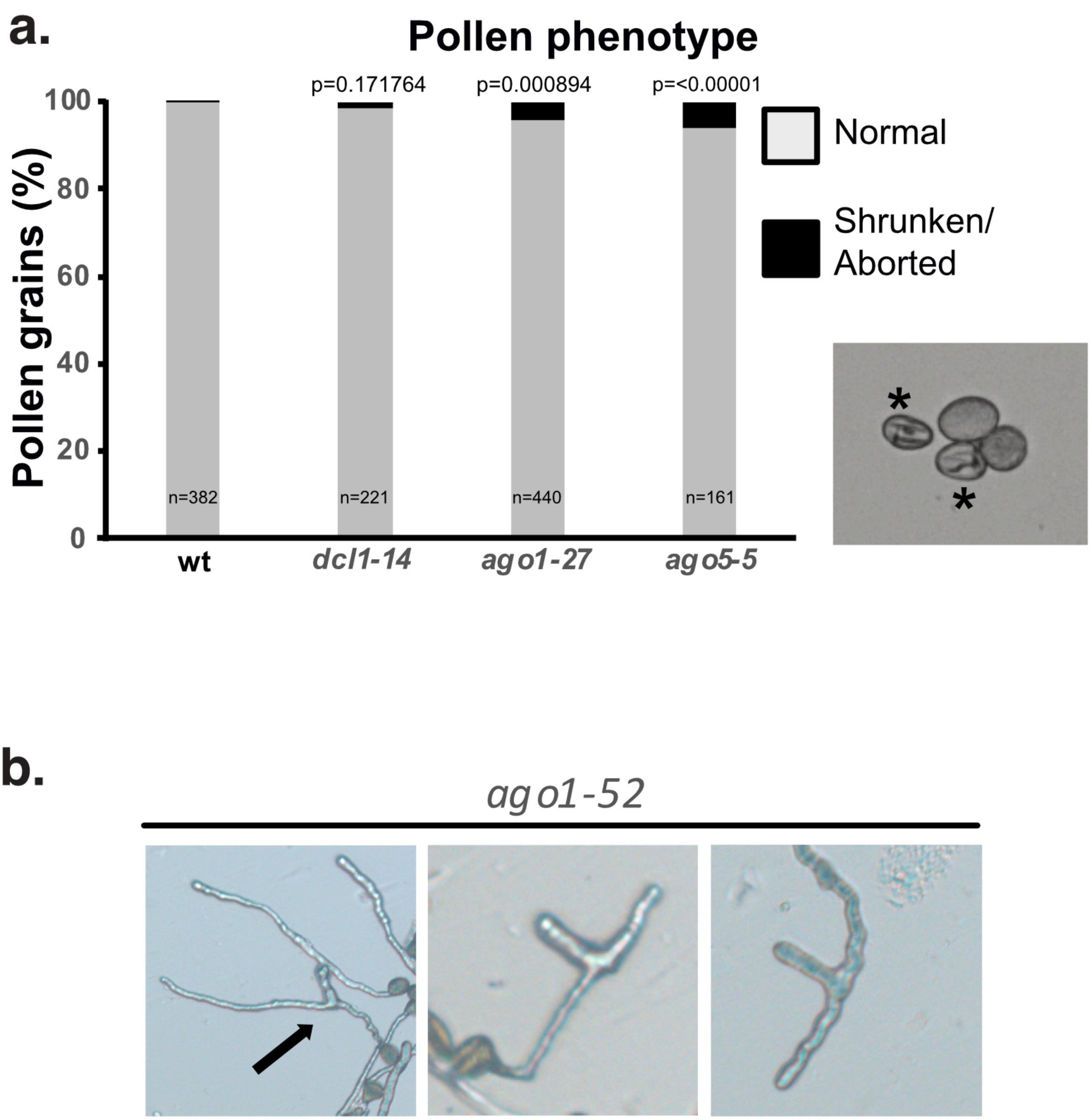

Supplemental Figure S3. Characterization of pollen phenotype in mutants for different components of miRNA pathway. Supports Figure 3. a) Analysis of pollen phenotype in Col-0 wild type (wt), *dcl1-14*, *ago1-27* and *ago5-5*. A representative picture showing two shrunken/aborted (marked with an asterisk) and two normal pollen grain is shown. A Chi-squared test was used to calculate p-values. b) Representative pictures of abnormal branched pollen tubes in *ago1-52*.

Supplemental Figure S4

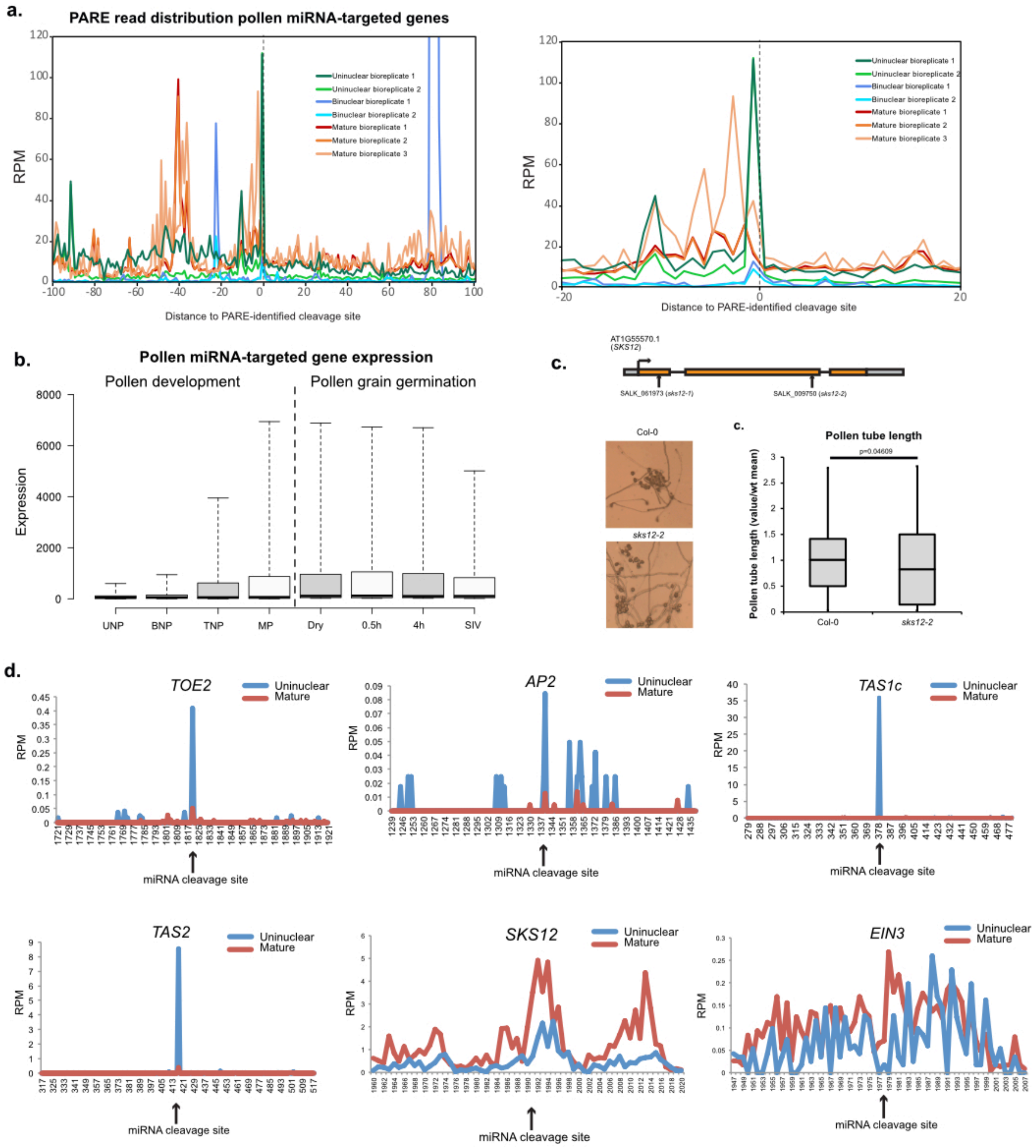

Supplemental Figure S4. Overview of miRNA gene-cleavage activity during pollen maturation. Supports Figure 4. a) Distribution of 5' ends of PARE reads for each bioreplicate analyzed around the predicted cleavage site (located at coordinate 0 in the X axis) in a 100 nt window. Grey zone represents the physical position covered by the bound miRNA. b) Level of expression of miRNA-targeted genes during pollen development and pollen grain germination present in the ATH1 microarray (UNP= uninuclear pollen, BNP=binuclear pollen, TNP= trinuclear pollen and MP= mature pollen grain, Dry= Desiccated mature pollen, 0.5h= In vitro-germinated pollen grains after 30 minutes, 4h= In vitro-germinated pollen grains after 4 hours and SIV= Pollen tubes grown through the stigma and style). Whiskers in the box plots extent to the 5th and 95th percentile. c) Diagram showing the location of the T-DNA insertion for the *sks12-1* (SALK_061973) and *sks12-2* (SALK_009750) mutants analyzed in this study. Representative pictures of pollen grain germination for wt and the *sks12-2* mutant. Length of the pollen tube for the genotypes indicated. P value is the result of a standard t-test with 2 tails and unequal variance. Whiskers in the box plots extent to the maximum and minimum values. d) PARE read distribution for uninuclear and mature pollen along miRNA target sites for representative miRNA-targeted genes in Arabidopsis: miR172-targeted genes *TOE2* and *AP2* and miR173-targeted *TAS1c* and *TAS2* and the miRNA-targeted genes analyzed here: *SKS12* and *EIN3*.

Supplemental Figure S5

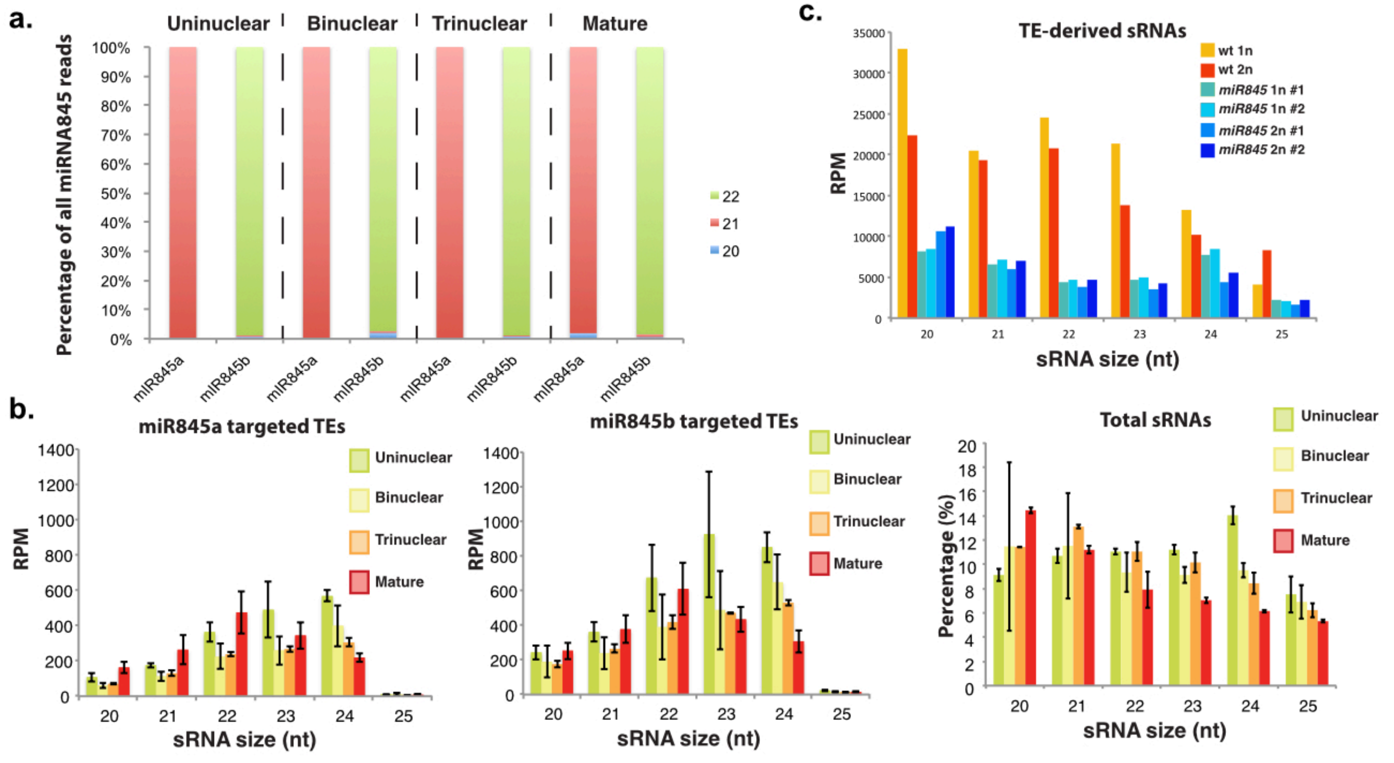

Supplemental Figure S5. Analysis of miR845 family members and target TEs during pollen development. Supports Figure 5. a) Preferential sRNA size for miR845a and b during pollen development. b) Accumulation profile of miR845a- and miR845b-targeted TEs. c) Accumulation profile in mature pollen grains of TE-derived sRNAs in wt and a miR845 mutant. 1n and 2n indicate the different number of ploidy of the samples analyzed.

Supplemental Figure S6

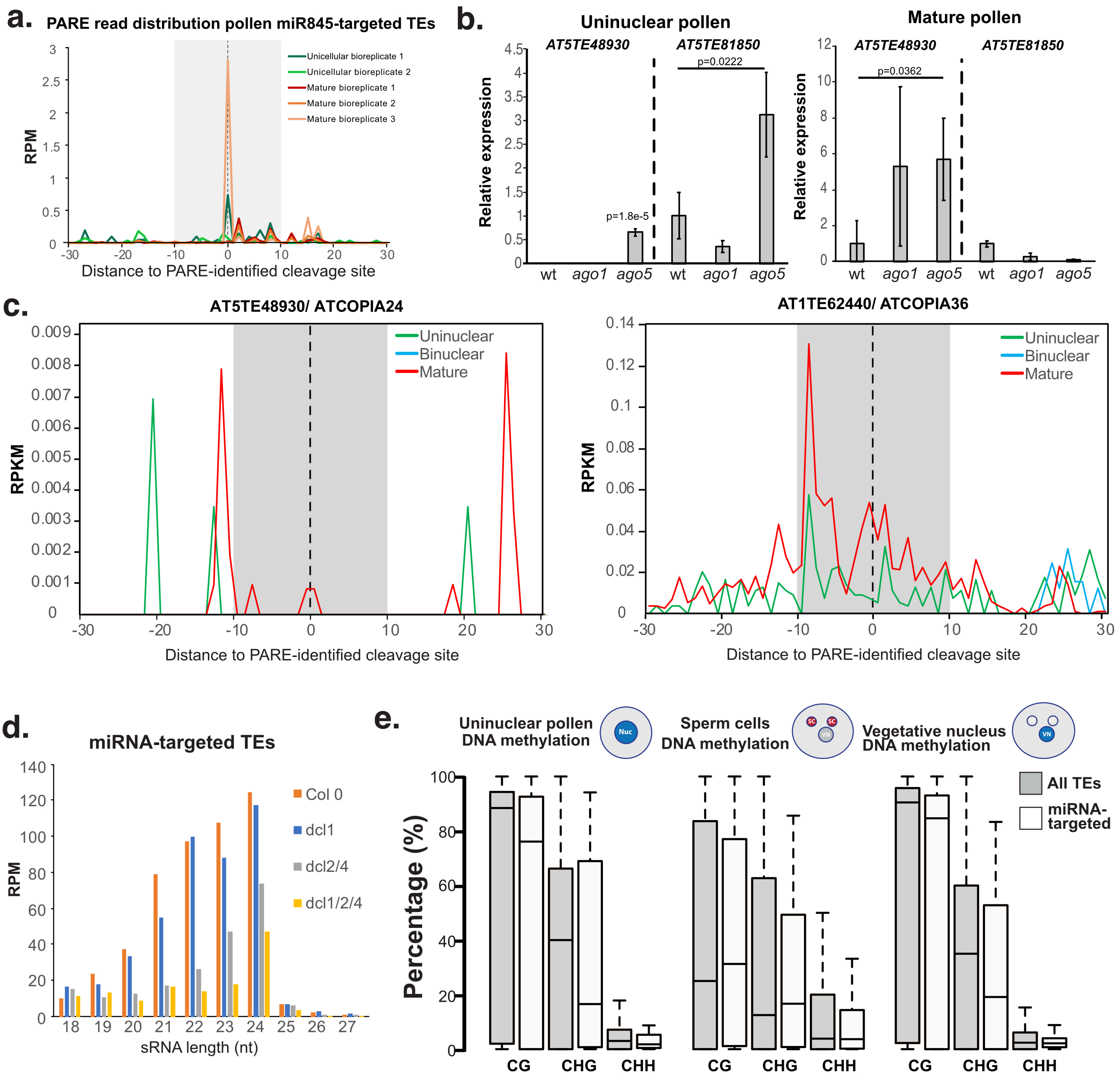

Supplemental Figure S6. Overview of miRNA TE-cleavage activity during pollen maturation. Supports Figure 6. a) Distribution of 5' ends of PARE reads for each bioreplicate analyzed around the predicted cleavage site for miR845 family members (located at coordinate 0 in the X axis) in a 30 nt window. Grey zone represents the physical position covered by the bound miRNA. b) Analysis of mRNA expression in uninuclear and mature pollen by RT-qPCR for two representative miRNA-targeted TEs (*AT5TE48930* and *AT5TE81850*) in wt, *ago1* and *ago5* mutants. Error bars represent the standard deviation values for the three bioreplicates analyzed. P value is the result of a standard t-test with 2 tails and unequal variance. c) Distribution of 5' ends of PARE reads for two miR845-targeted TEs (*AT5TE48930* and *AT1TE62440*) in a 30 nt window. Grey zone represents the physical position covered by the bound miRNA. d) Accumulation profile of sRNA derived from miRNA-targeted TEs in wt, *dcl1*, *dcl2/4* and *dcl1/2/4* datasets from Borges et al (2018). e) Levels of cytosine methylation for the different contexts (CG, CHG and CHH) in the uninuclear pollen, SCs and VN for all TEs (white boxes) or miRNA-targeted TEs (grey boxes). Significance was analyzed using a Mann Whitney test.

Supplemental Table S1

Library	Tissue of origin	Number of raw reads	Number of mapped reads	Associated figure/s
Uninuclear pollen bioreplicate 1	Uninuclear pollen	9,300,122	6,109,787	Figures 1, 5 and 6
Uninuclear pollen bioreplicate 2	Uninuclear pollen	8,500,128	5,896,816	Figures 1, 5 and 6
Binuclear pollen bioreplicate 1	Binuclear pollen	5,440,748	3,070,930	Figures 1, 5 and 6
Binuclear pollen bioreplicate 2	Binuclear pollen	9,482,338	7,567,948	Figures 1, 5 and 6
Trinuclear pollen bioreplicate 1	Trinuclear pollen	5,295,545	3,359,340	Figures 1, 5 and 6
Trinuclear pollen bioreplicate 2	Trinuclear pollen	6,418,752	5,140,866	Figures 1, 5 and 6
Mature pollen bioreplicate 1	Mature Pollen	2,850,419	2,579,710	Figures 1, 5 and 6
Mature pollen bioreplicate 2	Mature Pollen	2,403,201	2,171,869	Figures 1, 5 and 6
Uninuclear pollen PARE bioreplicate 1	Uninuclear pollen	28,251,329	25,364,673	Figures 4 and 6
Uninuclear pollen PARE bioreplicate 2	Uninuclear pollen	20,343,794	17,591,398	Figures 4 and 6
Binuclear pollen PARE bioreplicate 1	Binuclear pollen	6,675,395	6,095,362	Figures 4 and 6
Binuclear pollen PARE bioreplicate 2	Binuclear pollen	8,643,743	8,104,441	Figures 4 and 6
Mature pollen PARE bioreplicate 1	Mature Pollen	68,249,995	66,038,780	Figures 4 and 6
Mature pollen PARE bioreplicate 2	Mature Pollen	80,271,906	76,454,813	Figures 4 and 6
Mature pollen PARE bioreplicate 3	Mature Pollen	43,789,877	42,394,258	Figures 4 and 6
Mature leaf PARE	Mature leaf	11,069,341	8,500,913	Figure 4
AGO1 IP	Mature Pollen	7,284,899	6,554,226	Figure 2 and 5
AGO1 Input	Mature Pollen	793,318	686,289	Figure 2 and 5
AGO5 IP	Mature Pollen	4,391,748	3,376,420	Figure 2 and 5
AGO5 Input	Mature Pollen	1,398,543	1,197,703	Figure 2 and 5
Uninuclear pollen Input biorepicate 1	Uninuclear pollen	2,452,800	375,551	Figure 2 and 5
Uninuclear pollen Input biorepicate 1	Uninuclear pollen	7,649,757	450,810	Figure 2 and 5
AGO1 IP bioreplicate 1	Uninuclear pollen	8,216,216	2,533,762	Figure 2 and 5
AGO1 IP bioreplicate 2	Uninuclear pollen	1,226,318	388,914	Figure 2 and 5
AGO5 IP bioreplicate 1	Uninuclear pollen	2,568,975	1,058,543	Figure 2 and 5
AGO5 IP bioreplicate 2	Uninuclear pollen	369,091	102,804	Figure 2 and 5

Supplemental Table S1. Libraries produced in this study.

Supplemental Table S2

Target	miRNAs	UNM	BNP	MPG	UNM	BCP	MPG
AT1G03730	miR156						
SPL11	miR156						
SPL10	miR156						
AT1G69170	miR156						
SPL3	miR156						
AT2G40620	miR156						
SPL5	miR156						
SPL15	miR156						
AGD13	miR156						
SPL2	miR156						
SPL13A	miR156						
SPL13B	miR156						
SPL9	miR157						
AGL18	miR157						
AT1G62860	miR158						
AT5G16650	miR158						
AT1G18990	miR159						
MYB101	miR159						
AT2G34010	miR159						
MYB65	miR159						
SDG14	miR159						
MYB33	miR159						
OPT1	miR159						
ARF17	miR160						
ARF10	miR160						
ARF16	miR160						
AT1G63400	miR161.2						
ANAC021	miR164						
ANAC080	miR164						
ANAC092	miR164						
ANAC100	miR164						
PHV	miR165						
ATHB15	miR165						
ATHB14	miR165						
REV	miR165						
ARF6	miR167						
ARF8	miR167						
AGO1	miR168						
AT5G17780	miR168						
NF-YA5	miR169						
NF-YA3	miR169						
NF-YA2	miR169						
NF-YA10	miR169						
HAM1	miR171						
HAM2	miR171						
HAM3	miR171						
TOE1	miR172						
SNZ	miR172						
BZIP34	miR172						
AT3G18740	miR172						
AP2	miR172						
TOE2	miR172						
TOE3	miR172						
TAS1A	miR173						
TAS1C	miR173						
TAS2	miR173						
AT4G11680	miR2939						
AT1G50990	miR391						
AFB3	miR393						
AT3G23690	miR393						
AFB2	miR393						
TIR1	miR393						
GRH1	miR393						
AT1G27340	miR394						
GUN5	miR395						
GRF1	miR396						
GRF3	miR396						
GRF8	miR396						
GRF2	miR396						
AT1G19500	miR397						
SCE1	miR397						
AT5G02244	miR397						
AT5G18420	miR397						
AT3G15640	miR398						
AT1G06580	miR400						
AT1G62720	miR400						
AT2G06095	miR401						
AGO2	miR403						
UTR1	miR407						
AT4G01670	miR407						
AT2G47020	miR408						
AT5G03850	miR420						
AT5G02570	miR4243						
RS41	miR447						
AT3G26860	miR472						
TLP7	miR5013						
EIN3	miR5015						
UVH6	miR5021						
HMGB3	miR5021						
SKS12	miR5021						
AT1G71970	miR5021						
AT2G40520	miR5021						
AT3G15140	miR5021						
DEL1	miR5021						
AT3G50910	miR5021						
AT4G32590	miR5021						
AT5G10946	miR5021						
MIOX4	miR5630						
AT3G02555	miR5633						
CCT	miR5633						
AT1G38790	miR5643						
FLA3	miR5644						
CWINV2	miR5644						
AT4G31430	miR5646						
CPuORF17	miR5651						
PAO2	miR5651						
AT5G07410	miR5654						
AT5G27660	miR5657						
AT1G63057	miR5658						
BGAL11	miR5661						
ATSRL1	miR5998						
AT2G40116	miR5999						
TPR10	miR777						
SUVH6	miR778						
AT4G14810	miR780.1						
AT1G04290	miR782						
AT3G25290	miR8165						
TUA2	miR8167						
AT3G51940	miR8173						
AT2G23120	miR8176						
AT1G12250	miR822						
AT1G63060	miR823						
CMT3	miR823						
AGL16	miR824						
AOP2	miR826						
AT5G49640	miR826						
ZAC	miR829						
AT1G79245	miR830						
AT4G17550	miR834						
AT2G23900	miR845						
RCN1	miR846						
AT5G58375	miR846						
SAMDC	miR854						
AT1G48040	miR856						
AT1G24800	miR859						
AT1G24881	miR859						
AT1G25055	miR859						
AT1G25150	miR859						
AT1G25211	miR859						
AT2G17830	miR859						
AT5G16500	miR859						
DES-1-LIKE	miR863						
EXPA4	miR865						

Bioreplicates
0 1 2/3

Reads at target site
<1% >10%

Supplemental Table S2. High-confidence identified miRNA-targeted genes in PARE libraries. Target gene and its associated miRNA are indicated together with a heatmap of the presence of the target site in the different number of unicellular or mature PARE libraries in different shades of gray, and the level of accumulation of PARE reads in a 20 nt window surrounding the miRNA target site compared to the total accumulation of PARE reads for that gene for each individual bioreplicate studied with a blue-red gradient.

Supplemental Table S3

Target	Figure	Forward primer sequence	Reverse primer sequence
Northern blot of miRNA 161.1	Figure 1	ACCCCGATGTAGTCACTTTCA	
Northern blot of miR156	Figure 1	GTGCTCACTCTCTTCTGTCA	
Northern blot of miR845a	Figure 1	CATCAATTGGTATCAGAGCCG	
Northern blot of miR845b	Figure 1	CATCAATTTGGTATCAGAGCGA	
Northern blot control U6 snRNA	Figure 1	TCATCCTTGCGCAGGGGCCA	
qPCR UBQ10 (housekeeping gene)	Figure 3	AAGCAGTTGGAGGATGGCAGAAC	CGGAGCCTGAGAACAAGATGAAGG
qPCR EIN3	Figure 3	AACTGGCATGTCCACATCGAGAC	ATGAAACCTGGATGGTGCTGCTC
qPCR SKS12	Figure 3	ACGAGAGGAGTGTCCAGTCTTG	CTCAACCGCTACAGCGAAGAAG
KRP6 promoter In-Fusion cloned to drive P19 expression to the VN	Figure 3	CCGCCCCCTTCACCGAGCTCTCGTTGTTGCATCAGTCACTTAATTATTAC	ATAGCTCGTTCCATGAGCTCTCTCTCTTGGATTTTTGTGTGCTCTC
P19 cloning with In-Fusion site for insertion of KRP6 promoter	Figure 3	CACCGGTACCATGGAACGAGCTATACAAGG	CTCGCTTTCTTTTTCGAAGG
Genotyping sks12-1 SALK_061973	Figure 5	CGTCATTAATCTTCCAAGCCC	CATCTTCAGGGTCAGCGTAAG

Supplemental Table S3. Primers used in this study.

Supplemental Table S4

GEO accession	Data type	Tissue of origin	Associated figure/s
GSE6162	Transcriptomic ATH1	Pollen development	Figure 4
GSE17343	Transcriptomic ATH1	Pollen germination	Figure 4
GSE40501	Whole-genome bisulfite sequencing	Unicellular pollen, vegetative nuclei and sperm nuclei	Supplementary figure 6
GSE84122	High-throughput sequencing of sRNAs	1n and 2n mature pollen grains	Supplementary figure 5
GSE106117	High-throughput sequencing of sRNAs	miR845 mutant 1n and 2n mature pollen grain	Supplementary figure 5
GSE106117	High-throughput sequencing of sRNAs	wt, *dcl1, dcl2/4, dcl1/2/4* mature pollen grain	Supplementary figure 6
GSE79414	High-throughput sequencing of sRNAs	Leaves and root	Supplementary figure 1

Supplemental Table S4. Publicly available data analyzed in this study.
